# Numerical simulation of vascular tumour growth under antiangiogenic treatment: addressing the paradigm of single-agent bevacizumab therapy with the use of experimental data

**DOI:** 10.1186/s13062-016-0114-9

**Published:** 2016-03-22

**Authors:** Katerina D. Argyri, Dimitra D. Dionysiou, Fay D. Misichroni, Georgios S. Stamatakos

**Affiliations:** In Silico Oncology and In Silico Medicine Group, Laboratory of Microwaves and Fiber Optics, Institute of Communication and Computer Systems, School of Electrical and Computer Engineering, National Technical University of Athens, 9 Iroon Polytechniou, Zografos, GR 157 80 Athens, Greece

**Keywords:** Vascular tumour growth, Continuous model, Dynamical system, Antiangiogenic treatment, In silico oncology, Computational oncology

## Abstract

**Background:**

Antiangiogenic agents have been recently added to the oncological armamentarium with bevacizumab probably being the most popular representative in current clinical practice. The elucidation of the mode of action of these agents is a prerequisite for personalized prediction of antiangiogenic treatment response and selection of patients who may benefit from this kind of therapy. To this end, having used as a basis a preexisting continuous vascular tumour growth model which addresses the targeted nature of antiangiogenic treatment, we present a paper characterized by the following three features. First, the integration of a two-compartmental bevacizumab specific pharmacokinetic module into the core of the aforementioned preexisting model. Second, its mathematical modification in order to reproduce the asymptotic behaviour of tumour volume in the theoretical case of a total destruction of tumour neovasculature. Third, the exploitation of a range of published animal datasets pertaining to antitumour efficacy of bevacizumab on various tumour types (breast, lung, head and neck, colon).

**Results:**

Results for both the unperturbed growth and the treatment module reveal qualitative similarities with experimental observations establishing the biologically acceptable behaviour of the model. The dynamics of the untreated tumour has been studied via a parameter analysis, revealing the role of each relevant input parameter to tumour evolution. The combined effect of endogenous proangiogenic and antiangiogenic factors on the angiogenic potential of a tumour is also studied, in order to capture the dynamics of molecular competition between the two key-players of tumoural angiogenesis. The adopted methodology also allows accounting for the newly recognized direct antitumour effect of the specific agent.

**Conclusions:**

Interesting observations have been made, suggesting a potential size-dependent tumour response to different treatment modalities and determining the relative timing of cytotoxic versus antiangiogenic agents administration. Insight into the comparative effectiveness of different antiangiogenic treatment strategies is revealed. The results of a series of *in vivo* experiments in mice bearing diverse types of tumours (breast, lung, head and neck, colon) and treated with bevacizumab are successfully reproduced, supporting thus the validity of the underlying model.

**Reviewers:**

This article was reviewed by L. Hanin, T. Radivoyevitch and L. Edler.

## Background

Solid tumours progress through two separable phases: the avascular and the subsequent vascular one. In the initial phase that a tumour goes through, nutrients and oxygen are delivered to the tumour cells via diffusion processes alone from surrounding host capillaries. While in this phase, the tumour can be supported to grow to a diameter of just a few millimetres (1–2 mm) corresponding to a population threshold of 10^6^ cancer cells. The tumour could remain at this critical size, where cell proliferation is balanced by cell death, for a period of several months or even years without causing any serious damage to the host [1]. This phase is exploitable by in vitro experimentation, where tumour cells are cultivated in the laboratory as three-dimensional spheroids [2].

Eventually, the tumour mass reaches the critical point of transition from avascular to vascular phase and develops an intrinsic blood supply network which allows it to grow further. At this point, tumour cells secrete angiogenic factors such as vascular endothelial growth factor (VEGF) in response to hypoxia and thus, the angiogenic activation process termed as the angiogenic switch, occurs. Endothelial cells are then triggered to secrete matrix metalloproteinases that facilitate cell migration and proliferation by degrading the basement membrane. Endothelial cells will proliferate and eventually, the tumour will be penetrated by vessels. Finally, maturation of several vessels will occur as a result of molecular competition between activators and inhibitors regulating angiogenesis [3, 4].

Experimental and clinical evidence suggest that tumour angiogenesis, i.e. the tumour-induced growth of new capillary blood vessels in the body from already existing vasculature, is a key process in tumour development and cancer invasion. In particular, once angiogenesis has obtained its goal, new vessels provide the tumour mass with nutrients and oxygen, which are clearly of vital importance for its survival and further growth. Hence, the tumour may soon reach a cancer cell population of the order of magnitude of 10^9^, give rise to the first symptoms [5, 6] and eventually form metastases in distant organs. Angiogenesis occurring in a physiological context, e.g. during early embryogenesis [7] as well as in the female reproductive system [8] is completely different in numerous aspects comparing to tumour – induced angiogenesis. The latter, does not tend to create mature and stable vessels able to provide normal blood supply [4, 9]. Instead, tumour vasculature is characterized by a complex and dysfunctional structure resulting in higher fractal dimension [4, 10].

The concept of antiangiogenic treatment was originally introduced by J. Folkman. It was based upon the idea that the growth of a tumour is strongly dependent on the amount of blood vessels that it induces to grow [11, 12]. However, it was not until the nineties that initial experimental results suggesting that angiogenesis blockage could result in tumour regression came up [13]. Hence, the original target of antiangiogenic treatment was to block the transfer of nutrients and oxygen to the tumour by destroying the tumour vasculature until all cancer cells starve to death. That is why it was believed that the specific kind of treatment would be able to cure the disease. Even though there is now enough evidence that this is not the case, several mechanisms of action of angiogenic inhibitors have been elucidated in order to explain their anticancer effect.

The present work focuses in simulating the antitumour effect of bevacizumab, probably the most popular representative of the wider family of antiangiogenic agents in current clinical practice. In particular, bevacizumab is a recombinant humanized monoclonal antibody that acts by binding to VEGF, i.e. the main mediator of tumour angiogenesis, thereby inhibiting its interaction with the corresponding VEGF receptors on the surface of endothelial cells. Thus, with VEGF being thought to play a central role in the formation of tumour metastases [14], bevacizumab is considered a promising strategy for treating metastatic sites [15]. Various studies have been sought in order to analyse the actual efficacy of bevacizumab treatment [16]. Bevacizumab is currently believed to have multiple effects in tumour vasculature:

i. It induces regression of existing tumour vasculature [17, 18],
ii. It inhibits the proliferation of endothelial cells, thus causing impairment of further vessel growth [19],
iii. It normalizes surviving tumour vasculature facilitating the delivery of chemotherapeutic agents to the tumour tissue [20–22]. In particular, the antiangiogenic treatment effect is believed to improve both functionally and morphologically the abnormal and chaotic structure of tumour vessels. This transient effect, known as vascular normalization, results in a more normal and organized vasculature network. It is considered of vital significance since a major issue of chemotherapy treatment is the fact that a large percentage of the chemotherapeutic drug bypasses large areas of the tumour [23, 24], not being able to access all target sites due to the high abnormality of tumour vessels. Moreover, in the recent years, the scientific community has determined additional benefits of vascular normalization, such as the fact that it enhances radiosensitivity and tumour immunogenicity [25]. The latter is obtained via a better and easier access of leukocytes into the tumour parenchyma.

During the first years of the 21st century and up to this point in time much progress has been made towards developing mathematical models which describe malignant tumour growth while taking into account tumour induced angiogenesis explicitly [3, 26, 27], or even implicitly, as perturbator of the proliferation related parameters that characterize the tumour (through the modification of the probability of a newborn cell to re-enter the cell cycle in relation to the respective probability value in the largely necrotic layer of the tumour) [28–30].

A modelling approach considered to be a milestone in vascular tumour growth related literature due to its validity and minimal parameterization is the work by Hahnfeldt and colleagues, found in [31]. This model describes tumour growth under angiogenic signalling and to the best of our knowledge, it is the first model that incorporates a variable carrying capacity accounting for the time-dependent resources available to the tumour system. Following this work, many similar approaches have come up by investigating, generalizing, modifying [26, 32, 33] and/or extending the rationale suggested by Hahnfeldt et al. [31]. Various model extensions have been developed on this basis so as to distinguish between different subpopulations such as quiescent and cycling endothelial cells [34] as well as mature and immature endothelial cells [35]. Several approaches have also accounted for combination treatment consisting of the antiangiogenic agent in conjunction with radiotherapy [36, 37] or chemotherapy [10]. Additionally, it has been attempted to address chemotherapy resistance [38] as well as vascular pruning [39]. Another modification that is worth mentioning, is the one introduced by Bodnar and Forys [40] who have studied the impact of time delays on the model, for several versions of the initial modelling approach. Specifically, time delays were introduced in tumour growth as well as stimulation and inhibition of tumour vasculature. In many cases, a control theoretic setting has been selected, aiming at identifying optimal protocols of antiangiogenic regimens as well as combination treatment schemes [10, 36, 37, 41, 42]. Finally, Poleszczuk et al. [43], the work of whom is the basis for our modelling approach, have claimed that the model of Hahnfeldt et al. does not address the mode of action of antiangiogenic treatment acting on the signalling level. Instead, it addresses antivascular treatment effect acting cytotoxically on endothelial cells. Thus, they have modified a term of the initial model such that it simulates the actual mechanism of action of this kind of targeted therapy. More details on this work are provided in the Methods section along with the mathematical formulation of our approach.

To the best of our knowledge, the models developed up to this point in time, do not address the following issues. First of all, the standard bevacizumab mode of administration to human i.e. intravenous infusion. On the contrary, an instantaneous bolus is the standard assumption made [31, 43]. Additionally, according to the pertinent literature which is presented in the respective subsection, the one-compartment pharmacokinetic model which is the standard assumption in the existing models appears to be inferior to the more refined two-compartmental models that best describe bevacizumab pharmacokinetic properties. Pharmacokinetic constant values for an infusion two-compartmental bevacizumab pharmacokinetic model reported in literature, have been utilized. Secondly, the existing models do not appear to mathematically reproduce the asymptotic behaviour of a tumour volume in the theoretical case of total destruction of tumour neovasculature. Finally, to the best of our knowledge, the range of tumour types addressed by antiangiogenic models up to now has been very limited [44]. In order to broaden the range of tumour types addressed by the existing models for experimental fitting purposes we have exploited experimental data from four different tumour types (breast, lung, colon, head and neck).

The long term aim of our approach is to develop a specific Oncosimulator [45] based on a clinically adapted and validated multiscale model of vascular tumour growth and response to treatment. Certain models that have appeared in literature rely on preliminary validation based mostly on logical and mathematical tests and gross qualitative observations of experimental and/or clinical reality. Some other models, including the present work as well as previous work of our group [28, 46], also attempt a quantitative fitting to sets of experimental and clinical data [31, 44, 47]. Motivation to develop the present model has been provided by the large-scale European Commission funded project p-Medicine. Within the framework of this project, Oncosimulators [45] for the optimal personalized treatment of breast cancer patients treated with bevacizumab had to be developed.

The structure of the paper is the following: The Methods section consists of five subsections. Firstly, the continuous vascular tumour growth model stemming from the work in Poleszczuk et al. [43] is presented. Relevant equations are explained and model parameters involved are described. Secondly, details on the inclusion of bevacizumab pharmacokinetics are provided. Next, we elaborate on the numerical solution and implementation of the model and we demonstrate in a graphical way the information flow within the vascular tumour growth model under bevacizumab monotherapy model as a whole. The fourth subsection deals with the methodology of the conducted parameter analysis. Finally, there is one last subsection, outlining the methodology adopted for the fitting process. The Results and Discussion section includes three subsections. First, specific results revealing both free growth and tumour growth under bevacizumab monotherapy pattern are presented. Second, the parameter analysis results are shown, revealing the effect of model parameters involved on the dynamics of the biological system. Third, the results of the conducted fitting process are presented and discussed. Finally, conclusions are drawn.

## Methods

An early version of the vascular tumour growth model has already been outlined in [48]. A more analytical description, along with further results, parameter analysis and fitting of the model to actual experimental data will be presented in the following sections.

### A continuous approach to modelling the mechanism of action of antiangiogenic treatment applied on a vascularized tumour

The scientific problem addressed consists of three interdependent processes: tumour development, tumour angiogenesis and the antiangiogenic treatment effect. Thus, a mechanistic model monitoring both the tumour and vascular compartment while addressing the targeted nature of the specific kind of treatment could give valuable insight into antiangiogenic treatment mode of action. To the best of our knowledge, the first modelling approach meeting these requirements is the approach of [43], stemming from previous, much investigated work of [31]. We have used this approach as a basis in order to simulate the bevacizumab monotherapy effect and reproduce the results of a series of *in vivo* experiments in mice bearing diverse types of tumours (breast, lung, head and neck, colon).

Multiple biologically essential phenomena of cancer cell population dynamics are incorporated into the model: cancer cell proliferation and cancer cell death (through the assumption of Gompertzian growth), post-vascular dormancy (the state where tumour growth ceases due to a balance eventually achieved between proangiogenic and antiangiogenic factors), secretion of endogenous proangiogenic factors (such as VEGF, fibroblast growth factors, platelet-derived growth factor, angiopoietin-1 etc.) by the tumour, secretion of endogenous antiangiogenic factors (such as thrombospondin, angiostatin, endostatin, angiopoietin-2, etc.) by the tumour and natural endothelial cell loss. Finally, the model takes into account antiangiogenic treatment - induced endothelial cell death as well as resulting tumour cell death.

To this point it should be mentioned that strictly speaking, the model concerns monoclonal tumourigenesis. In particular, due to the ordinary differential equations (ODE) formalism which is valid only for homogeneous systems, the tumour could be theoretically viewed as consisting of a hypothetical clone with the average properties of all tumour cells in space. This approximation is justifiable, as in the vascular phase in which the model is applicable, the number of cells is big enough for the system to be viewed as homogeneous. For the same reason, the behaviour of the cancer cells can be considered deterministic.

The dynamical system described in [43] is governed by a pair of ODEs which reflect the interplay between tumour volume (*V*) and carrying capacity (*K*). Before we proceed to present the equations involved in the vascular tumour growth model, the primary assumptions on which the model rests need to be formulated:

i. The tumour has spherical symmetry,
ii. The diffusion process via which the aforementioned factors are transported is in a quasi-stationary state,
iii. The concentration of the stimulator is a radially symmetric, continuously differentiable function,
iv. The clearance rate of proangiogenic factors is a monotonically increasing function of drug concentration and it is always greater than the respective value in the absence of treatment,
v. The change of drug concentration inside the tumour caused by the dysfunctional vasculature is governed by the proportionality to a bounded and decreasing function of tumour volume.

For the sake of completeness, a few remarks regarding the mathematical formulation of Hahnfeldt et al. model should be mentioned as this was the starting point and the basis for a constantly expanding family of vascular tumour growth models, including both [43] and the work presented in this article. As it was also mentioned in the previous section, the model of Hahnfeldt et al. assumed Gompertzian growth of tumour volume *V*(*t*) without however following the classical approach. Instead, they have introduced a time-dependent carrying capacity *K*(*t*) and a dependence of the rate of change of *K* (*dK/dt*) on *K*, *V,* and *t*.

The initial basis on which Hahnfeldt et al. [31] set up the equation describing the rate of change of carrying capacity was the one formulated below:

*Rate of change of carrying capacity* ***=*** *(spontaneous loss of functional vasculature)* ***+*** *(stimulatory capacity of the tumour)* ***+*** *(endogenous inhibition of existing vasculature)* ***+*** *(impairing of tumour vasculature due to administered angiogenic inhibitors).*

The first term reflecting the intrinsic loss rate was assumed to be proportional to carrying capacity, while the last one was taken to be a typical treatment-induced death term proportional both to carrying capacity and drug concentration. The second and third terms, reflecting stimulatory and inhibitory action induced by tumour cells respectively, were computed by applying a diffusion-consumption equation for the concentration of stimulators and inhibitors. Assuming that the tumour is in quasi-steady state and radial symmetry as well, the partial differential equation (PDE) was reduced to an ODE with the stimulator/inhibitor concentration as the unknown function of the distance from the center of the tumour. Subsequently, Hahnfeldt et al. approximated the solution for sufficiently small clearance rate (inhibitor case) and sufficiently large clearance rate (stimulator case), concluding that the inhibitor term should tend to grow at a rate *K*^*α*^ ⋅ *V*^*β*^ faster than the stimulator term, where *α* + *β* ≈ 2/3. Finally, following thorough fitting of the model to experimental data, they concluded that the term reflecting spontaneous loss of functional vasculature is negligible.

In 2011, Poleszczuk et al. [43] have omitted the term of Hahnfeldt et al. that reflected angiogenic inhibition due to administered inhibitors, claiming that it actually reflected antivascular treatment acting cytotoxically on endothelial cells. Instead, they have modified the term describing the endogenous angiogenic stimulatory capacity of the tumour accordingly, so as to account for the antiangiogenic treatment effect that acts on the signalling level by moderating endogenous angiogenesis stimulation of the tumour. In particular, they have reapplied the diffusion-consumption equation for the concentration of stimulators by perturbing the clearance rate by a function of antiangiogenic drug concentration. Hence, they have mathematically formulated the dependence of the extent of angiogenic stimulation and thus the dependence of tumour volume on the amount of drug in the host.

Following this modification, the model of Poleszczuk et al. [43] reads:

1 $$ \frac{dV}{dt}=-{\lambda}_1\cdot V\cdot \ln \left(\frac{V}{K}\right) $$

2 $$ \frac{dK}{dt}=-{\lambda}_2\cdot K+c\cdot \frac{\left(\beta +{V}^p\right)\cdot V}{\alpha \cdot \left(\beta +{V}^p\right)+I(t)}-d\cdot K\cdot {V}^{2/3} $$

where *V* stands for tumour volume, *K* for carrying capacity, *I*(*t*) for the antiangiogenic drug concentration and λ_1_, λ_2_, *c*, *d*, *β*, *α* and *p*, for parameters explained in Table 1. All coefficients are non-negative, except for proportionality constant *c* where only positive values are allowed in order to preclude the biologically irrelevant behaviour of an untreated tumour with self-regressing carrying capacity.

**Table 1:** Description of the variables and parameters used in the vascular tumour growth and the two-compartmental pharmacokinetic bevacizumab models

Mathematical Symbol	Description	Units	Value^a^	Reference^a^
Vascular tumour growth model				
*t*	Time	day	n/a	-
*V*	Tumour volume	mm^3^	n/a	-
*K*	Tumour carrying capacity	mm^3^	n/a	-
*λ*_1_	Gompertzian growth constant	day^−1^	0.192	[31, 43]
*λ*_2_	Proportionality constant related to the natural mortality of endothelial cells	day^−1^	0	[31, 43]
*c*	Proportionality constant related to the term reflecting endogenous stimulation of the tumour upon the vasculature	mg/(day · mm^3*p*^ · kg)	5.85	[31, 43]
*d*	Proportionality constant related to the term reflecting endogenous inhibition of tumour vasculature	day^−1^ · mm^−2^	0.00873	[31]
*α*	Parameter reflecting the stimulator clearance rate	mg/(mm^3*p*^ · kg)	1	[43]
*β*	Parameter reflecting the extent of the abnormal phenotype of tumour vasculature	mm^3*p*^	1	[43]
*p*	Parameter reflecting the extent of the abnormal phenotype of tumour vasculature	-	0	[43]
*I*	Drug concentration in plasma	mg/kg	n/a	-
Two-compartmental pharmacokinetic bevacizumab model				
*t*	Time-point	day	n/a	-
*I*	Bevacizumab concentration in plasma	mg/ml	n/a	-
*n*	Number of infusions to be administered	-	9	[60]
*D*	Dose	mg	dosage*weight	-
*T*	Infusion duration	day	1/48	[66]
*V*_*c*_	Volume of central compartment	ml	7.975	[64]
*k*_12_	Transfer constant from central to peripheral compartment	day^−1^	0.7536	[64]
*k*_21_	Transfer constant from peripheral to central compartment	day^−1^	0.3144	[64]
*k*_*e*_	Rate of elimination	day^−1^	0.3888	[64]
*d*	Dosage	mg/kg	5	[60, 61, 65]
*w*	Host’s weight	kg	0.025	[60, 64]
*t*_*D*_	Administration time-point of anti-angiogenic treatment	day	t_1_ = 1	-
			t_2_ = 4	
			t_3_ = 8	
			t_4_ = 11	
			t_5_ = 15	
			t_6_ = 18	
			t_7_ = 22	
			t_8_ = 25	
			t_9_ = 29	

^a^Used for indicative free growth and/or Intermittent bevacizumab monotherapy simulations

Prompted by a remark made by Dr. Leonid Hanin in his capacity as a reviewer of the present manuscript, we have proceeded to a correction of the asymptotic behaviour of the system as the drug concentration tends to infinity (*I*(*t*) → ∞). According to our initial gross assumption and in agreement with the asymptotic behaviour of the ODE model developed by [43], the tumour volume tends to zero as *I*(*t*) → ∞. This appears to be acceptable as a first approximation. However, we have adopted Hanin’s suggestion to take into account the refined mathematical observation that as *I*(*t*) → ∞ the tumour volume should tend to V*, with V* corresponding to the critical avascular tumour volume which is reached when the angiogenic switch occurs.

More specifically, based on the widely acknowledged mechanism of action of antiangiogenic agents, the expected behaviour of the system as the drug concentration tends to infinity (*I*(*t*) → ∞) would be to tend to a state (V*, K*) where V* as above and K*, represents the carrying capacity corresponding to the tumour while in the avascular phase. Note that K* = V* since carrying capacity is defined as the maximal tumour volume that can be sustained using the current resources.

However, when the antiangiogenic drug concentration is sufficiently high (*I*(*t*) → ∞) the dynamical system takes the following form:

3 $$ \frac{dV}{dt}=-{\lambda}_1\cdot V\cdot \ln \left(\frac{V}{K}\right) $$

4 $$ \frac{dK}{dt}=-{\lambda}_2\cdot K-d\cdot K\cdot {V}^{2/3} $$

According to Eq. (4) the carrying capacity has become a decreasing function, eventually turning the tumour volume into a decreasing function as well. This will finally drive the whole system to state (0, 0).

To address this apparent discrepancy we have substituted (*V*, *K*) for (*V*-V*, *K*-V*) preserving the structure of the model as it was such that (*V*-V*, *K*-V*) → (0, 0) and finally, (*V*, *K*) → (V*, V*). Of course, this intervention slightly modifies the underlying assumptions as well. Specifically, the updated equations suggest that the total amount of tumour-induced angiogenic stimulators does not actually depend on the entire tumour volume *V* but instead, on the part of tumour volume that has been developed following the angiogenic switch i.e. on the quantity V-V*. This is biologically plausible, if one takes into account that the angiogenic switch, when the tumour has already reached the critical size V*, is actually the moment at which the tumour begins to overexpress angiogenic stimulators [49]. In other words, once in the vascular phase during which the model is applicable, the part of the tumour volume developed prior to the triggering of the angiogenic switch does not contribute to the secretion of proangiogenic factors and subsequently to the growth of carrying capacity. Similarly, angiogenic inhibitors are assumed depend on the quantities V-V* and K-V*.

Hence, we have:

$$ \begin{array}{c}\hfill \frac{d\left(V-{V}^{*}\right)}{dt}=-{\lambda}_1\cdot \left(V-{V}^{*}\right)\cdot \ln \left(\frac{V-{V}^{*}}{K-{V}^{*}}\right)\hfill \\ {}\hfill \frac{d\left(K-{V}^{*}\right)}{dt}=-{\lambda}_2\cdot \left(K-{V}^{*}\right)+c\cdot \frac{\left(\beta +{\left(V-{V}^{*}\right)}^p\right)\cdot \left(V-{V}^{*}\right)}{\alpha \cdot \left(\beta +{\left(V-{V}^{*}\right)}^p\right)+I(t)}-d\cdot \left(K-{V}^{*}\right)\cdot {\left(V-{V}^{*}\right)}^{2/3}\hfill \end{array} $$

Finally, we concluded to the following ODE system monitoring the rate of change of both the total tumour volume *V*(*t*) (i.e. prevascular plus vascularized) and the total carrying capacity (i.e. adjacent and tumour-induced),

5 $$ \frac{dV}{dt}=-{\lambda}_1\cdot \left(V-{V}^{*}\right)\cdot \ln \left(\frac{V-{V}^{*}}{K-{V}^{*}}\right) $$

6 $$ \frac{dK}{dt}=-{\lambda}_2\cdot \left(K-{V}^{*}\right)+c\cdot \frac{\left(\beta +{\left(V-{V}^{*}\right)}^p\right)\cdot \left(V-{V}^{*}\right)}{\alpha \cdot \left(\beta +{\left(V-{V}^{*}\right)}^p\right)+I(t)}-d\cdot \left(K-{V}^{*}\right)\cdot {\left(V-{V}^{*}\right)}^{\raisebox{1ex}{$2$}\!\left/ \!\raisebox{-1ex}{$3$}\right.} $$

For the case of untreated growth where *I*(*t*) = 0 the dynamical system becomes:

7 $$ \frac{dV}{dt}=-{\lambda}_1\cdot \left(V-{V}^{*}\right)\cdot \ln \left(\frac{V-{V}^{*}}{K-{V}^{*}}\right) $$

8 $$ \frac{dK}{dt}=-{\lambda}_2\cdot \left(K-{V}^{*}\right)+\frac{c}{\alpha}\cdot \left(V-{V}^{*}\right)-d\cdot \left(K-{V}^{*}\right)\cdot {\left(V-{V}^{*}\right)}^{\raisebox{1ex}{$2$}\!\left/ \!\raisebox{-1ex}{$3$}\right.} $$

Note that for the rest of this article, as in both [31] and [43], we will omit the first term of eqs. (6) and (8), reflecting the spontaneous loss of functional vasculature. ODE system consisted of eqs. (7) and (8), captures the well acknowledged tumour growth slowdown to a plateau size, the value of which is determined by the eventual balance between angiogenic stimulators and inhibitors. In particular, the steady state solution of the system is:

9 $$ {\mathrm{V}}_{\mathrm{SS}}={V}^{*}+{\left(\frac{c}{\alpha \cdot d}\right)}^{3/2} $$

suggesting that only parameters involved in stimulator and inhibitor term modulate the plateau value of tumour volume.

Regarding the value assigned to the threshold of avascular tumour volume V* throughout this work, we have considered a threshold of tumour diameter equal to 1 mm. Indeed, relevant literature suggests that avascular tumours in humans are limited by a maximal size of 1–2 mm of diameter [50, 51] and presents biological evidence that avascular tumours of mice do not grow beyond 1 mm of diameter [52]. Assuming spheroid shape, this limit size corresponds to a tumour volume equal to 0.52 mm^3^. Finally, in accordance with [43], it is imposed that parameter *α* is fixed to a value of one (mg/(mm^3p^ · kg)) throughout this work. In the future, further investigation on the role of parameter *α* is needed as it could enable the simulation of different tumours with common untreated growth timecourse and diverse responses to antiangiogenic treatment. This feature could serve as a vehicle to addressing mechanisms like intrinsic tumour resistance to antiangiogenic treatment [53].

### Inclusion of bevacizumab pharmacokinetic properties

This section outlines the computation of bevacizumab concentration *I*(*t*) at a given timepoint *t*. This function is involved in the right-hand side of Eq. (6) and more specifically, in the term reflecting the angiogenic stimulatory capacity of the tumour.

**Fig. 1:**
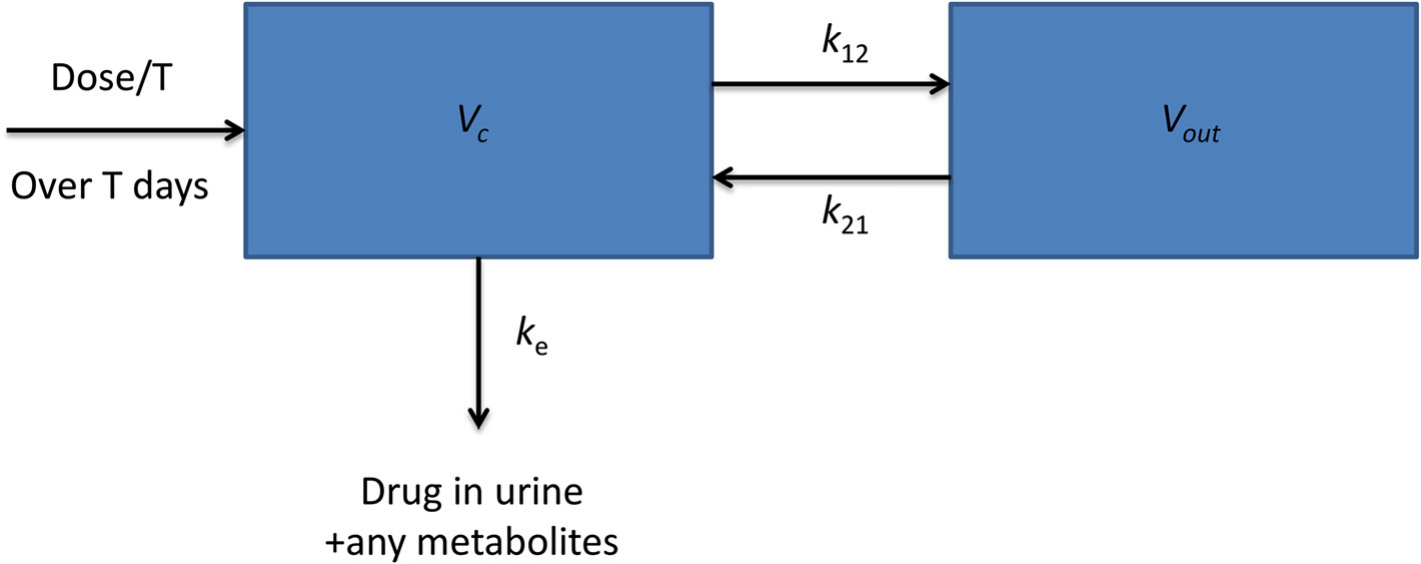
Schematic representation of the two – compartmental pharmacokinetic model. *k*_*a*_ absorption rate constant for the case of intravenous route of administration (intravenous infusion); *V*_*c*_, volume of central compartment; *V*_*out*_, volume of distribution of outer compartment; *k*_*12*_, distribution rate constant from central compartment to outer compartment; *k*_*21*_, distribution rate constant from outer compartment to central compartment; *k*_*e*_, elimination rate constant

Regarding the pharmacokinetic properties of bevacizumab, a large scale review of pertinent literature has been conducted. Two-compartmental models assuming first-order elimination appear to give the best description of bevacizumab pharmacokinetic data [54–56]. Hence, we proceeded with the implementation of a two-compartmental pharmacokinetic model (Fig. 1). Taking into account that the specific antiangiogenic agent is administered to human patients via the intravenous route, the case of intravenous infusion has been addressed by applying zero-order absorption, reflecting steady drug delivery into the patient’s systemic circulation.

The central compartment represents blood and all highly perfused tissues, i.e. vital organs that are in rapid equilibrium with blood such as lungs, kidney and liver. Drug elimination is assumed to occur from the central compartment according to the first order transfer rate *k*_*e*_ since kidney and liver are the two most important clearing organs. The outer compartment corresponds to poorly perfused tissues.

Given the fact that monitoring drug concentration at the anatomical region of interest is not feasible, the concentration of drug in plasma is typically assumed to reflect the concentration in the site of the target tissue. Hence, the equation characterizing the central compartment is the one to be introduced in the vascular tumour growth model.

Thus, if *T* stands for duration of infusion, *D* for administered dose and *t*_*D*_ for timepoint of drug administration, the function that describes bevacizumab concentration *I*(*t*) at a given timepoint *t* in plasma following a single drug infusion, is mathematically formulated as below:

While the infusion takes place, i.e. while *t* − *t*_*D*_ ≤ *T*:

10 $$ I(t)=\frac{D}{T}\cdot \left[\frac{A}{a}\left(1-{e^{-a}}^{\left(t-{t}_D\right)}\right)+\frac{B}{b}\left(1-{e}^{-b\left(t-{t}_D\right)}\right)\right] $$

During the post-infusion period, namely while *T* < *t* − *t*_*D*_:

11 $$ I(t)=\frac{D}{T}\cdot \left[\frac{A}{a}\left(1-{e}^{-aT}\right)\cdot {e}^{-a\left(t-{t}_D-T\right)}+\frac{B}{b}\left(1-{e}^{-bT}\right)\cdot {e}^{-b\left(t-{t}_D-T\right)}\right] $$

where,

12 $$ a=\frac{k_{21}\cdot {k}_e}{b} $$

13 $$ b=0.5\left({k}_{12}+{k}_{21}+{k}_e-\sqrt{{\left({k}_{12}+{k}_{21}+{k}_e\right)}^2}-4{k}_{21}\cdot {k}_e\right) $$

14 $$ A=\frac{1}{V_c}\frac{a-{k}_{21}}{a-b} $$

15 $$ B=\frac{1}{V_c}\frac{b-{k}_{21}}{b-a} $$

Further information on the model assumptions and governing equations can be found in [57]. In order to compute the pharmacokinetic effect of multiple drug doses, all drug concentration curves are internally (in the code) super-positioned at each simulation timepoint so as to sum the contribution of current drug dose with the one of previous administrations. Parameters and variables involved in the pharmacokinetic model are explained in Table 1.

In order to comply with the necessary uniformity of units and given that the drug concentration is monitored in plasma, we have internally divided the output of the implemented two-compartmental model (in units of mg/ml) by the typical value of blood plasma density (in kg/ml) [58].

### Implementation and numerical solution

We proceeded with the implementation of the model in MATLAB. The details concerning the principle m-files are listed and explained below.

#### Function drug concentration

This function implements the two-compartmental pharmacokinetic model for administration via intravenous infusion (as is the case for bevacizumab use in clinical practice). It calculates the concentration of the antiangiogenic agent at each timepoint also taking into account the contribution of all previous infusions.

#### Function vascular tumour growth

This function computes the derivatives involved in Eqs. (5)-(6), which describe vascular tumour growth.

#### Script vascular tumour growth_main

Given the.csv file containing the parameter values, the specific script file resolves the problem with the solver ode45 which implements a Runge Kutta method with a variable time step for efficient computation. It also plots simultaneously the variables *V* and *K* (representing tumour volume and carrying capacity, respectively) as functions of time.

**Fig. 2:**
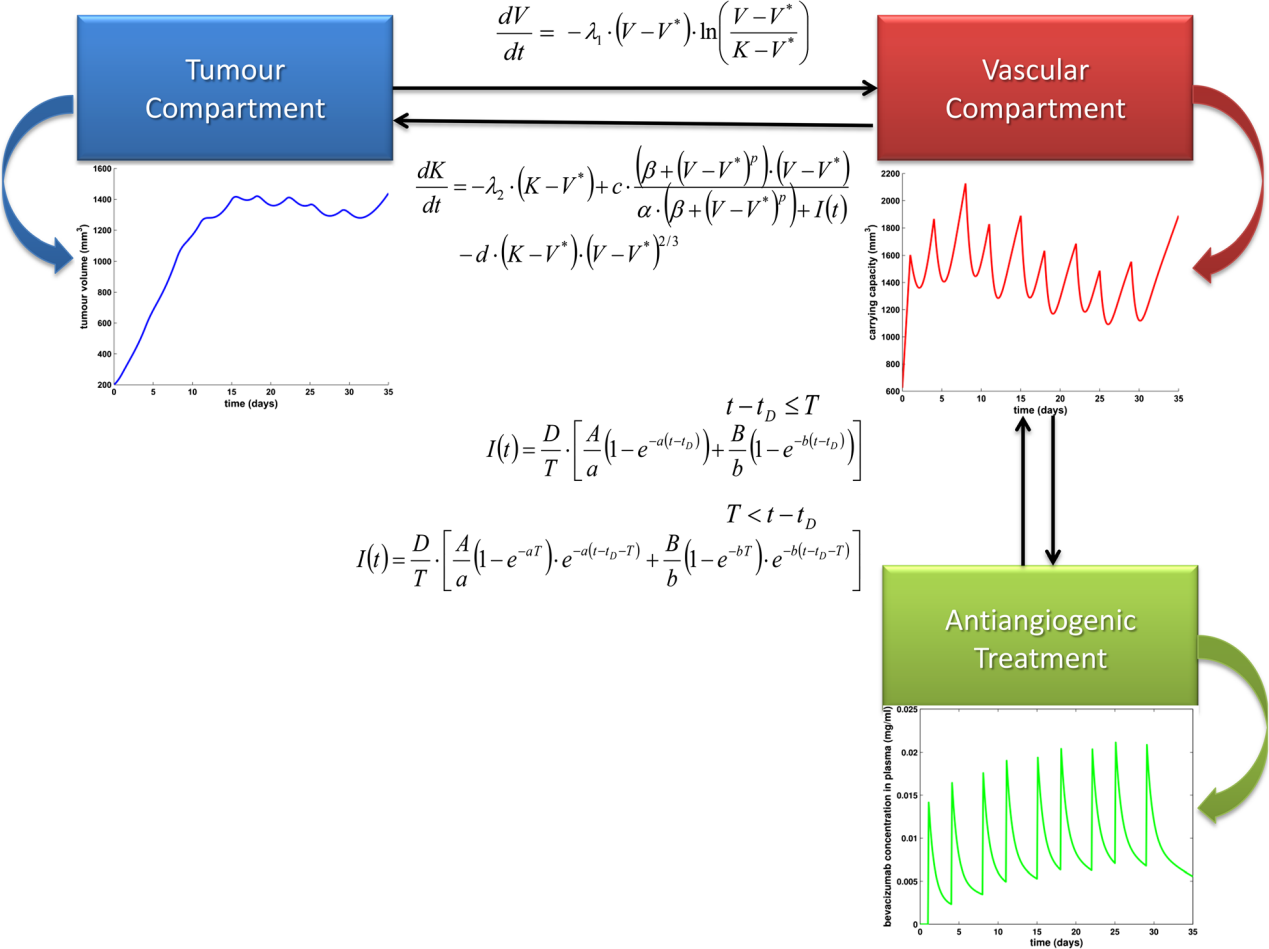
Information flow inside the vascular tumour growth under antiangiogenic treatment model. Parameters are explained in Table 1

It is worth noting that the experiments concerning free tumour growth as well as tumour growth under intermittent bevacizumab treatment have been performed on a desktop computer with an AMD Phenom(tm) II X6 1055 T Processor 2.80 GHz, 8.00GB RAM in Windows 7 with 64-bit operating system and that the code execution time is of the order of a few seconds. Figure 2 demonstrates the information flow inside the vascular tumour growth model.

### An initial step towards investigating the impact of input parameters to the model output, for an untreated vascularized tumour

An initial exploratory set of parametric simulations has been conducted in order to investigate the impact of the input factors on the output of the model as well as to identify the model parameters exerting the greatest influence on the model predictions. This has been a two-stage process: initially, a varying one parameter at a time approach has been selected so as to study the dynamic behaviour of an untreated solid tumour. The effect of perturbing each free growth -related factor on the output of the model when all other parameters are kept constant at the reference values [31] has been studied in order to gain insight into the role of each input parameter to untreated tumour evolution. Although perturbations of the reference value by diverse percentages have been applied (±5 %, ±10 %, ±20 %, ±50 %), the results that came up applying a perturbation percentage equal to 20 % are indicatively presented. The impact of the input parameters has been measured as reflected by their effect on the value of the plateau reached by the tumour (in mm^3^), as well as the timing of its attainment (in days). As a second step, the sensitivity of the parameters that drive the angiogenic control of the tumour has been studied with respect to the attained value of tumour volume plateau. Their combined effect on the plateau values is visualized in three-dimensional space. The input parameters to be varied are λ_1_, *c* and *d*.

For the needs of parameter analysis, the following is considered:

i. The tumour has reached the plateau when the whole number part of the value of tumour volume coincides with the respective one of carrying capacity for at least ten consecutive simulation time-steps.
ii. The timepoint that corresponds to the first of the aforementioned time steps is the exact time of plateau and the repeated value is the value of the plateau.

The aforementioned endpoints reflect the dynamics of the untreated disease. Were the parameter investigation conducted in the context of the therapy module, extra endpoints should also be selected, i.e. endpoints interpretable as efficacy measures of the administered antiangiogenic treatment, such as tumour growth inhibition or tumour volume reduction.

### Reproducing the outcome of a series of *in vivo* experiments in mice treated with bevacizumab

Given the fact that the effects of bevacizumab monotherapy were not found to be significantly dependent on whether the drug is intravenously or intraperitoneally administered [59], we proceed with the reproduction of the results of a series of *in vivo* experiments in mice intraperitoneally treated with bevacizumab. In particular, eight *in vivo* experiments conducted in mice bearing breast (KPL-4 cell line), lung (H226 cell line), head and neck (SCC1 cell line) as well as colon (HCT116, HT29, HCP40 and HP40 cell lines) tumour xenografts have been selected from relevant literature [60–62] in order to fit the model to actual experimental data. These experiments aimed at examining the antitumour activity of bevacizumab monotherapy and combination treatment. Of course, in the latter case, datasets corresponding to unperturbed tumour growth and bevacizumab monotherapy arm alone were exploitable by the model.

For the needs of fitting the model to the available data, two extra m-files have been implemented. In particular, a cost function has been defined as the difference between the model prediction and the actual value of the tumour volume, for the available instances of experimental data. The goal of the fitting process is to minimize the objective function, in order to obtain the most accurate estimation of the variables under study. A second function has been implemented so as to specify the parameter values that minimize the squared value of the objective function of each data-point. We have used Μatlab built-in function for solving nonlinear least-squares (lsqnonlin), in conjunction with the trust region reflective algorithm.

In order to quantify the experimental data obtained through relevant literature [60–62], we have utilized PlotDigitizer software which allows data extraction (mean value ± standard deviation) via its digitization. Assuming that the effect of bevacizumab administration would perturb the parameter values that yield the simulation of the untreated dataset, or at least some of them, the fitting process for each experimental dataset (control group and treatment group) has been conducted in two steps: for each experiment, firstly, fitting of the free growth module to the control group data and subsequently, based on the result of the previous parameter estimation, fitting of the respective bevacizumab monotherapy module to the treatment group data. In cases where multiple treatment groups were included in the same experiment (i.e. in experiments 3 and 4 where there exists one treatment group per administered dose level), the fitting of bevacizumab monotherapy module to the treatment group data for a specific dose level was initiated based on the result of the parameter estimation corresponding to the nearest lower dose level treatment group. Next, the adopted methodology is outlined.

#### Fitting the free growth module

Local fitting has been performed with respect to four parameters involved in the unperturbed vascular tumour growth module (λ_1_, *c*, *d*, *K*_0_), with *K*_0_ representing the initial carrying capacity.

#### Initial point

The least squares solver requires a user-supplied initial point (λ_1_^in^, *c*^in^, *d*^in^, *K*_0_^in^) to start from. The values of the first three components are set in accordance with [31]. The value of parameter *K*_0_^in^ is selected such that for each dataset the ratio (*V*_0_/*K*_0_^in^) is equal to the corresponding ratio in [31], where *V*_0_ is set equal to the value of the first available experimental measurement of tumour volume for the specific dataset. This choice allows us to mimic a realistic tumour dynamics for mice in accordance with relevant literature.

#### Imposed bound constraints

These specify the region of parameter space where the least squares solver will search for the locally (i.e. around the selected initial point) optimal solution. The value of the first available experimental measurement of tumour volume for each dataset has been assigned to the lower boundary of parameter *K*_0_. This choice prevents the solver from selecting an optimal parameter value that would yield a biologically irrelevant behaviour such as an unperturbed tumour growth timecourse with an initial decreasing section. For the lower boundaries of the rest of parameters involved in the fitting process non-negativity has been imposed.

##### Fitting the bevacizumab monotherapy module

Local fitting has been performed with respect to five parameters involved in the vascular tumour growth under bevacizumab monotherapy module, namely (λ_1_, *c*, *β*, *p, K*_*0*_). Among those, *β* and *p* are the ones that are exclusively involved in the therapy-related module, reflecting the pathological structure of tumour vessel network and decreasing the drug concentration that actually accesses the tumour. Parameters λ_1_ and *c*, were selected based on the expected impact of treatment administration on the bioprocess that each one reflects. Thus, taking into account the mode of action of bevacizumab binding VEGF, parameter *c* (involved in the term representing endogenous stimulation of the tumour upon the vasculature) has been allowed to fluctuate. Moreover, by also allowing the decrease of λ_1_ which is the unique parameter that can affect tumour growth rate independently of the angiogenic compartment, the direct antitumour effect that anti-VEGF therapy may induce [63] is reflected. Given the fact that instantaneous treatment effect has been assumed as a first approximation, *K*_0_ is allowed to fluctuate for datasets where the first available experimental timepoint coincides with the first administration timepoint. Parameter *d*, reflecting the action of endogenous angiogenesis inhibitors, is considered independent of the bevacizumab monotherapy effect and as such, it remains fixed to the value that has been determined through the respective fitting of the unperturbed growth model. Values of parameters involved in the pharmacokinetic model for the case of host-mouse have been extracted from [64].

#### Initial point

As mentioned above, the least squares solver requires a user-supplied initial point (λ_1_^in^, *c*^in^, *β*^in^, *p*^in^, *K*_0_^in^) from which the search of the parameter space initiates. The values of λ_1_^in^, and *c*^in^ are the optimal values that were determined via the previous fitting process of the corresponding experiment. Parameter values *β*^in^ and *p*^in^ are adopted from [43]. Lacking any precise information, the initial value *K*_0_^in^, is calculated such that for each dataset the ratio (*V*_0_/*K*_0_^in^) is equal to the corresponding ratio that has been determined in the case of the control group, where *V*_0_ is set equal to the value of the first available experimental measurement of tumour volume for the treatment arm dataset. However, in several datasets, manual tuning of initial parameter values was necessary in order to achieve a better fitting result.

#### Imposed bound constraints

The upper boundary of λ_1_, i.e. the parameter that directly influences the tumour growth rate, has been set equal to the respective estimated value that was determined via the fitting of the free growth module in order to account for a potential direct antitumour effect of bevacizumab. For the rest of the parameters involved in the fitting process of the treatment module, non-negativity has been imposed.

## Results and discussion

### Indicative tumour growth patterns

In order to study the qualitative behaviour of the model, numerous sets of code executions have been conducted by testing the code with different parameter values, simulation time span, initial values and code module (free growth, constant treatment, intermittent treatment). Having adopted the classical graph representation, the following indicative results are presented so as to illustrate several points of interest concerning the dynamics of the model.

We have focused on the case of host-mouse due to the availability of reference values [31, 43] as well as the existence of relevant experimental data extracted from literature [60–62].

#### Free growth

**Fig. 3:**
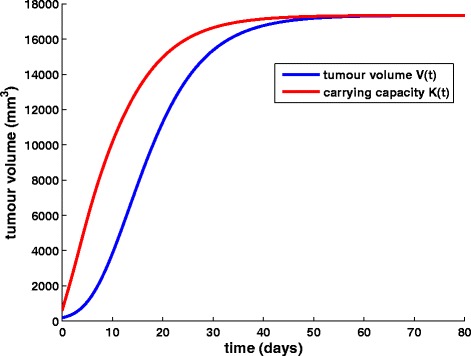
The free growth pattern. Simulation results for an untreated tumour characterized by the parameter values shown in Table 1

Figure 3 demonstrates the timecourse of tumour volume and carrying capacity for the free growth of the tumour characterized by the parameter values used by Hahnfeldt et al. [31]. Assuming spheroid shape, timepoint 0 corresponds to a tumour radius equal to 3.63 mm and thus, to a vascularized tumour. Growth slowdown is obvious and the asymptotic limit obtained is approximately equal to 17347 mm^3^, a value consistent with the one that comes up through the steady state solution (see Eq. (9)) as well as the results in [31]. This plateau is a theoretical concept, representing the upper horizontal asymptote that bounds the tumour volume never to be actually exceeded as the tumour burden would have already become lethal to the host [6].

#### Tumour growth under bevacizumab monotherapy

**Fig. 4:**
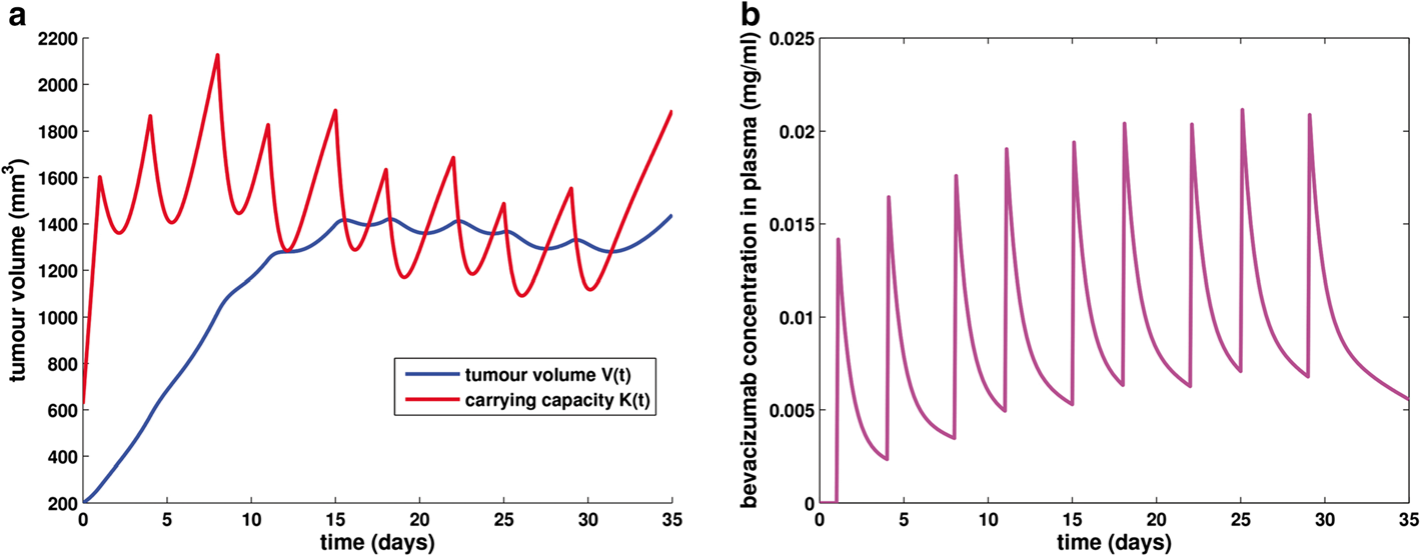
The growth pattern for a treated tumour and bevacizumab concentration timecourse in host’s plasma. **a** Simulation results for a tumour treated with bevacizumab. The regimen applied has been used in the context of various *in vivo* experiments in mice [60, 61, 65]. Parameter values are shown in Table 1. **b** The timecourse of bevacizumab concentration in plasma for the simulated treatment scheme, the details of which are shown in Table 1. Values of parameters involved in the pharmacokinetic model for the case of host-mouse were extracted from [64]

**Fig. 5:**
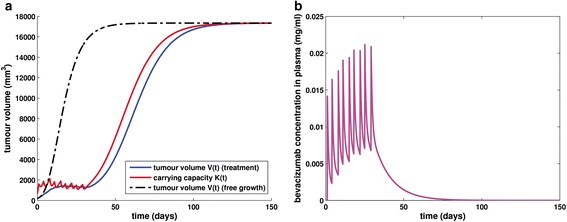
Free growth pattern versus treated tumour growth pattern. **a** Simulation results for a tumour treated with bevacizumab according to a scheme used in the context of various *in vivo* experiments in mice [60, 61, 65] and characterized by the parameter values shown in Table 1 for an extended simulation time duration (150 days). **b** The time course of bevacizumab concentration in plasma for the simulated treatment scheme, the details of which are shown in Table 1. Values of parameters involved in the pharmacokinetic model for the case of host-mouse were extracted from [64]

In order to demonstrate the qualitative behaviour of the treatment module (Figs. 4 and 5), we proceeded with simulating the effect of the application of a bevacizumab monotherapy treatment scheme to a mouse weighing 25 g, bearing a tumour characterized by parameter values extracted from [43], except for parameter *d* which was assigned to the value used in [31]. The simulated scheme consists of the administration of 5 mg/kg of bevacizumab as a single-agent, twice weekly, for a total of 9 doses. The specific treatment scheme details i.e. dosage, number and time interval between infusions, have been used in the context of various *in vivo* experiments in mice [60, 61, 65]. Parameter values involved in the two-compartmental pharmacokinetic model have been adopted from [64] and are also only applicable for the case of host-mouse. Regarding the duration of infusion, we have considered infusions lasting 30 minutes, i.e. the minimum value of the typical range of the standard bevacizumab infusion rate for human patients [66], due to the lack of relevant mice-specific information.

Treatment begins on the 1st day, when the tumour size has already grown to a volume equal to 269 mm^3^ i.e. when the tumour radius of is approximately equal to 4 mm assuming spheroid shape. Taking into account that timepoint 0 corresponds to a tumour of 200 mm^3^, it can be observed that an initial overshoot of tumour volume (*V*) occurs, due to the fact that the tumour has been so far untreated. With the start of the regimen administration, a sharp decrease of carrying capacity is obvious as well as the subsequent response of tumour volume. This pattern reflects the fact that antiangiogenic treatment targets directly the vascular compartment and through that, the tumour compartment responds. However, it is worth noting that the specific tumour is not responsive in terms of treatment-induced tumour shrinkage but only in terms of tumour growth inhibition, i.e. comparing to the tumour evolution in the case of untreated growth (Fig. 5a). A closer inspection of Fig. 4a, reveals that each time the curve *K*(*t*) intersects curve *V*(*t*), the monotonicity of function *V*(*t*) alters. This behaviour that can be easily noticed from Eq. (3) reflects the fact that carrying capacity is actually defined as the maximal tumour volume that the current vascular system can support. Figure 4b shows the time evolution of bevacizumab concentration in plasma as computed via the two-compartmental pharmacokinetic model.

Extending simulation time enables the study of the treatment impact on the attainment of the upper limit that constitutes the plateau of the tumour, namely, it allows the study of the treatment effect in its entirety. Hence, the numerical simulation has been extended to a simulation time period equal to 150 days. The respective results suggest that the treatment administration has not affected the value of the plateau, yet it has caused a considerable growth delay of 85 days (Fig. 5a).

The behaviour demonstrated in Fig. 5a provides clear indications of the temporary effect of antiangiogenic treatment, being however able to delay tumour evolution. In particular, a tumour growth inhibition by 19.38 % can be observed, computed as the percentage of change in tumour volume in the treatment module simulation with respect to the free growth module simulation, at the end of the treatment (i.e. day 32).

### Parameter analysis results

#### Perturbing parameter *λ*_1_

**Fig. 6:**
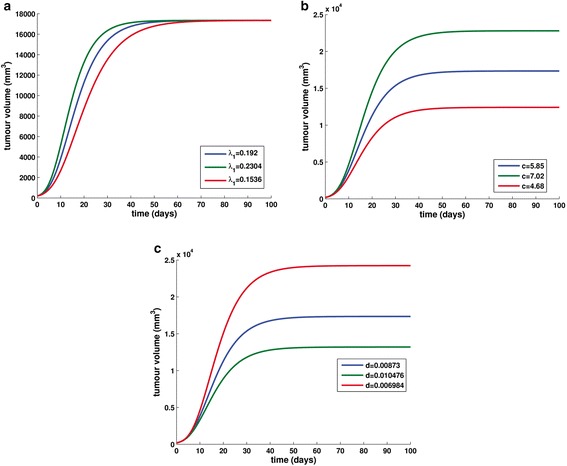
The effect of perturbing parameters *λ*_1_, *c* and *d* on the volume of the untreated tumour. **a**. *λ*_1_ is perturbed by ±20 % around its reference value [31] when all other parameters are kept constant at the reference values [31] on the volume of the untreated tumour. **b**. *c* is perturbed by ±20 % around its reference value [31] when all other parameters are kept constant at the reference values [31] and **c**. *d* is perturbed by ±20 % around its reference value [31], when all other parameters are kept constant at the reference values adopted in [31]

Equation (7) shows that the Gompertzian growth constant *λ*_1_ is the only parameter that affects tumour growth rate independently of the angiogenic compartment, making it thus a direct modulator of the doubling time characterizing the simulated tumour at each timepoint. All parameters have been kept constant, while *λ*_1_ has been perturbed by a uniform 20 per cent either side of the reference value (Fig. 6a) which has been set equal to 0.192 day^−1^ [31]. Hence, values 0.192 day^−1^, 0.2304 day^−1^ and 0.1536 day^−1^ have been assigned to *λ*_1_. Taking Eq. (7) into account, it is expected that for untreated disease, higher values of *λ*_1_ would result in a more aggressive tumour. Indeed, as demonstrated in Fig. 6a, increasing the value of *λ*_1_ leads to a faster growing tumour, while decreasing the value of *λ*_1_ has the opposite effect. From the viewpoint of the selected endpoints, a closer inspection of Fig. 6a reveals the impact of parameter *λ*_1_ to the actual timepoint that the tumour attains the plateau. On the contrary, as it is also expected based on Eq. (9), the specific parameter does not affect the value of the plateau. The measured effect is presented in Table 2.

**Table 2:** Effect of the variation of one input parameter value at a time, on the value of the tumour plateau as well as on the timepoint of its attainment

Parameter values			Units	Time of plateau (day)	Value of plateau (mm^3^)	Variation of plateau value (%)
*λ*_1_	Reference value	0.192	day^−1^	95	17347	n/a
	+20 %	0.2304		79	17347	0
	−20 %	0.1536		118	17347	0
*c*	Reference value	5.85	day^−1^	95	17347	n/a
	+20 %	7.02		96	22803	+31.45 %
	−20 %	4.68		101	12413	−28.44 %
*d*	Reference value	0.00873	day^−1^ · mm^−2^	95	17347	n/a
	+20 %	0.010476		87	13196	−23.93 %
	−20 %	0.006984		99	24243	+39.75 %

All results regarding variation of plateau value are in agreement with the respective calculations based on Eq. (9)

#### Perturbing parameter *c*

All other parameters have been kept constant, while *c*, reflecting the stimulatory capacity of the tumour upon the inducible vasculature, has been perturbed by a uniform 20 percent either side of the reference value (Fig. 6b), which was set equal to 5.85 day^−1^ [31]. Values 5.85 day^−1^, 7.02 day^−1^ and 4.68 day^−1^, were thus assigned to *c*. According to Fig. 6b an increased value of parameter *c* would result in higher angiogenic stimulatory capacity of the tumour and thus to its faster growing, as expected based on Eq. (8). Similarly, decreasing *c* would result in a slower growing tumour, a conclusion that is also corroborated in a graphical way (Fig. 6b). The great impact of perturbing *c* in the actual value of the plateau attained by the tumour is presented in Table 2.

#### Perturbing parameter *d*

All parameters remain constant, while *d*, reflecting endogenous inhibition of further angiogenesis, has been perturbed by a uniform 20 percent either side of the reference value (Fig. 6c), which was set equal to 0.00873 day^−1^ · mm^−2^ [31]. Thus, values 0.00873 day^−1^ · mm^−2^, 0.010476 day^−1^ · mm^−2^ and 0.006984 day^−1^ · mm^−2^ have been assigned to *d*. It is expected that high values of parameter *d* lead to tumours with high endogenous angiogenesis inhibition and hence, to slower tumour growth. In a similar way, lower values of *d* are expected to correspond to tumours with less angiogenesis inhibition and thus, to faster growing tumours. In accordance with the previous statement, Fig. 6c displays the timecourse of a slower growing tumour attaining a lower plateau for the maximal value assigned to *d* and the time course of a faster growing tumour attaining a higher plateau for the minimal value assigned to *d*. The impact of perturbing *d* on the actual value of the plateau attained by the tumour is presented in Table 2.

As it has been expected based on Eq. (9) reading the mathematical formulation of steady state solution of the ODE system, Fig. 6 and Table 2 render *c* and *d*, i.e. the parameters related to the total amount of angiogenic stimulators and inhibitors respectively, the sole modulators of the value of the plateau attained by the tumour, while *λ*_1_ appears to be the main modulator of the timepoint at which the tumour reaches the plateau.

**Fig. 7:**
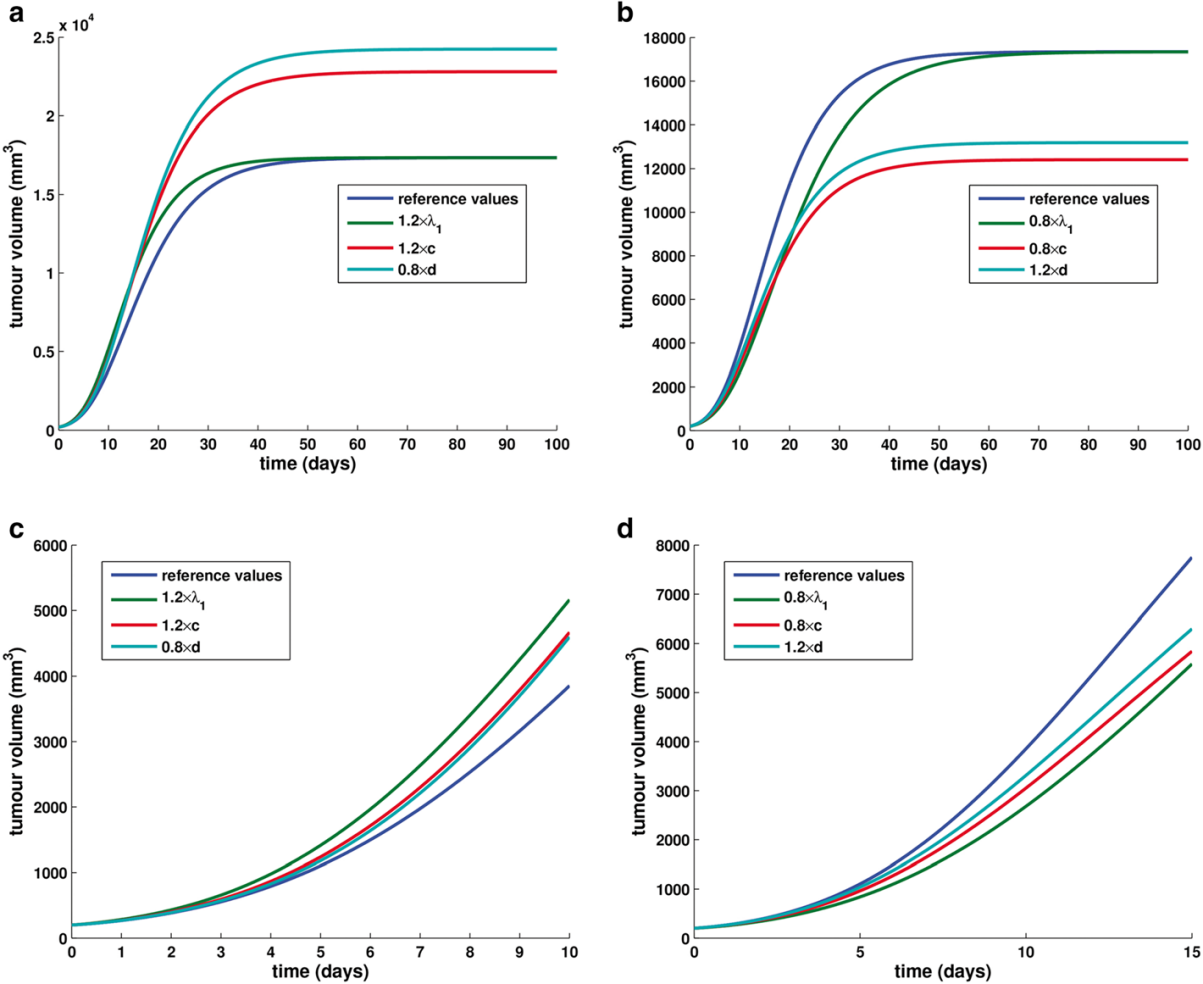
The shortest path to a more aggressive tumour and towards a less aggressive tumour. **a** The effect of perturbing *λ*_1_, *c* and *d* by 20 % in order to construct a more aggressive tumour. **b** The effect of perturbing *λ*_1_, *c* and *d* by 20 % in order to construct a less aggressive tumour. **c** Selection of an earlier end-point (day 10): the effect of perturbing *λ*_1_, *c* and *d* by 20 % in order to construct a more aggressive tumour. **d** Selection of an earlier end-point (day 15): the effect of perturbing *λ*_1_, *c* and *d* by 20 % in order to construct a less aggressive tumour

Two extra sets of simulations have been conducted in order to determine in a graphical way the “shortest path” to a more and a less aggressive tumour respectively, by varying one parameter at a time. It is noted that for convenience, the graphs corresponding to the different varied parameters are plotted in the same coordinate system for each study performed. Thus, we have proceeded with the simultaneous demonstration of the effect of perturbing *λ*_1_ and *c* by the percentage of +20 % and *d* by the percentage of −20 % of the respective reference values (Fig. 7a), so as to increase the aggressiveness of the tumour. Subsequently, we present the effect of perturbing *λ*_1_ and *c* by a uniform −20 % and *d* by a uniform +20 % of the respective reference values (Fig. 7b) in order to construct a less aggressive tumour.

The results that are presented with the use of graphical representation suggest that in the specific region of parameter space, *d*, the parameter that reflects the concentration of angiogenesis inhibitors is the most sensitive parameter to changes that create a more aggressive tumour, while *c*, the parameter that relates to angiogenic stimulatory capacity, seems to be the most sensitive parameter to changes that create a less aggressive tumour. This suggests that antiangiogenic treatment aiming at reducing the amount of angiogenic stimulators (e.g. treatment with antiangiogenic agent bevacizumab), could be a more efficient strategy than increasing the amount of angiogenic inhibitors in the tumour (e.g. administration of angiostatin). Of course, further investigation is needed for this claim to be corroborated.

A closer inspection of the two comparative graphs reveals a dependence of the sensitivity on the final timepoint considered. For example, if one considers a final timepoint in the initial phase of the tumour (e.g. day 10 in the “towards a more aggressive tumour” scenario and day 15 in the “towards a less aggressive tumour” scenario), *λ*_1_ appears to be the most influential input parameter (Fig. 7c, d). This behaviour suggests that in the early vascular phase, the primary factor driving the time evolution of the overall system is the intrinsic features of the tumour, reflected by parameter *λ*_1_ which is independent of the angiogenic compartment. However, an untreated tumour will eventually reach a point in time where it has become sufficiently large and the parameters that are related with the angiogenic process will become the most influential to the tumour development. Hence, in the latter case, the application of antiangiogenic treatment would be more efficient while in the former case, treatment methods that target directly the tumour compartment such as the typical chemotherapy agents acting cytotoxically on cancer cells would be probably more effective. Again, further investigation is necessary for the confirmation of this claim.

It is a well-recognized fact that the angiogenic potential of a tumour is regulated by a dynamic balance between endogenous proangiogenic and antiangiogenic factors. Therefore, a significant step would be to gain deeper insight into the quantitative effect of each one of the relevant parameters (*c* and *d* respectively) as well as their combined effect on the aggressiveness of the tumour as reflected by the value of the attained tumour plateau. To this end, we have made use of the equation giving the steady state solution of the unperturbed growth module of the dynamical system (Eq. (9)). This further study of the dependence of the plateau value attained by an untreated tumour on *c*, *d* (*λ*_1_ does not affect it) is of great significance since it could determine boundaries for the parameter values that yield a biologically realistic plateau for simulating actual clinical tumours. Based on Eq. (9), the value of tumour plateau can be considered as a function of parameter *c*, *a* or *d*, with the rest of parameters involved in the computation set equal to reference values. Thus, the effect of each one of these parameters on the value of tumour plateau can be studied by varying the value of parameter of interest. Regarding the range of parameter values used for the needs of parameter analysis (see Table 3), an upper bounded tumour volume plateau by an order of magnitude of 10^6^ (mm^3^) has been assumed in accordance with cases regarding mice tumours encountered in literature [31] as well as with the plateau value calculations from local fitting results to experimental tumour volume measurements [60–62] that are presented in the following section. A further constraint has been imposed by assuming that in the untreated growth context no self-regressing vasculature can occur and thus, carrying capacity is required to be an increasing function (yielding a lower boundary of *c* value and an upper boundary of *d* value).

**Fig. 8:**
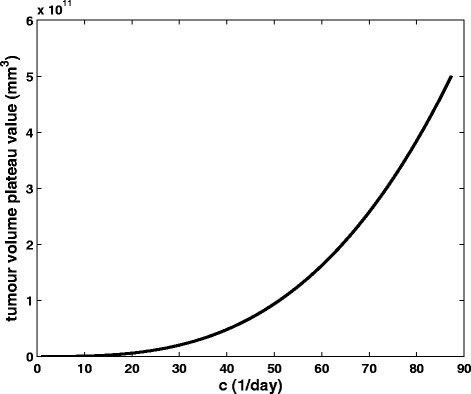
The effect of tumour angiogenesis stimulators on the tumour volume plateau. Graphical representation of the function describing the dependence of tumour volume plateau value on the parameter reflecting the impact of endogenous angiogenesis stimulators (*c*) on the growth rate of carrying capacity

Figure 8 shows the effect of parameter *c* on the tumour volume plateau. Once more, it is graphically corroborated that higher values of the parameter that reflects the endogenous angiogenic stimulation lead to higher tumour volume plateau values. The gradient of the tumour volume plateau function varies smoothly along the curve suggesting the non-existence of a specific area exhibiting greater sensitivity throughout the whole range of values considered for *c*.

**Fig. 9:**
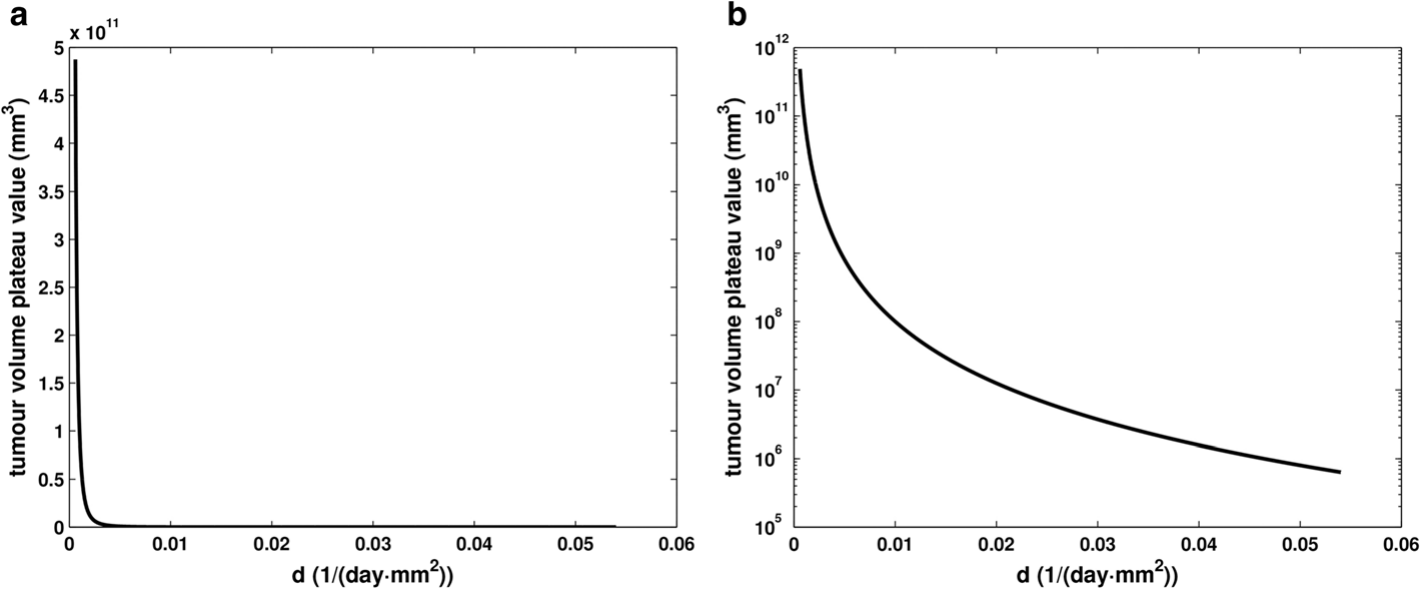
The effect of tumour angiogenesis inhibitors on the tumour volume plateau. Graphical representation of the function describing the dependence of tumour volume plateau value on the parameter reflecting the impact of endogenous angiogenesis inhibitors (*d*) on the growth rate of carrying capacity a. displayed in a logarithmic-linear scale b. displayed in a linear-linear scale

In sharp contrast, Fig. 9 demonstrates a more pronounced effect on the tumour volume plateau for smaller values of parameter *d,* reflecting the endogenous inhibition of angiogenesis. Indeed, as the initial steepness of the curve suggests (displayed in a logarithmic-linear scale in Fig. 9a and in linear-linear scale in Fig. 9b), a variation of parameter *d* over the initial 22.47 % of the domain of the function i.e. [0.000585, 0.012005] causes the tumour volume plateau size to span two orders of magnitude (i.e. approximately 66.67 % of the function’s range).

**Fig. 10:**
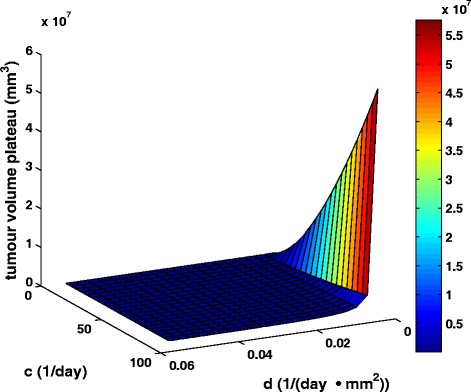
The combined effect of tumour angiogenesis stimulators and inhibitors on the tumour volume plateau. Three-dimensional visualization of the combined effect of parameter c reflecting the action of endogenous angiogenic stimulators and parameter *d* reflecting the action of endogenous angiogenic inhibitors on the value of the plateau attained by the tumour. Different colours correspond to distinct values of attained tumour volume plateau

Finally, in order to capture the dynamics of the molecular competition between endogenous proangiogenic and antiangiogenic factors in a tumour, we have proceeded with the three-dimensional visualization of the joint effect of parameters *c* and *d* on the value attained by the tumour volume plateau (Fig. 10).

At first glance, the counteracting effect of endogenous proangiogenic and antiangiogenic factors becomes obvious, with the most aggressive tumours (in terms of plateau value) being characterized by higher *c* values combined with lower *d* values. Virtual tumours with lower values of parameter *d* are more aggressive than those with higher *d* values. This difference remains approximately constant as *c* value increases. Similarly, tumours characterized by higher values of parameter *c* are more aggressive than those with lower *c* values with the difference remaining nearly constant over the entire *d* value range considered. Both parameters have a comparable effect on tumour value plateau throughout the range of values considered with the more pronounced one caused by parameter *c*. Finally, in accordance with the conclusions previously drawn (Figs. 8 and 9), the influence of parameter *c* retains the same character over the entire value range considered. By contrast, parameter *d* demonstrates a sensitive behaviour on the tumour volume plateau for smaller *d* values with the sensitivity decreasing as *d* values increase. The curves characterized by constant *d* = 0.00873 day^−1^ · mm^−2^ and constant *c* = 5.85 day^−1^ correspond to Figs. 8 and 9 respectively.

### Local fitting results

**Fig. 11:**
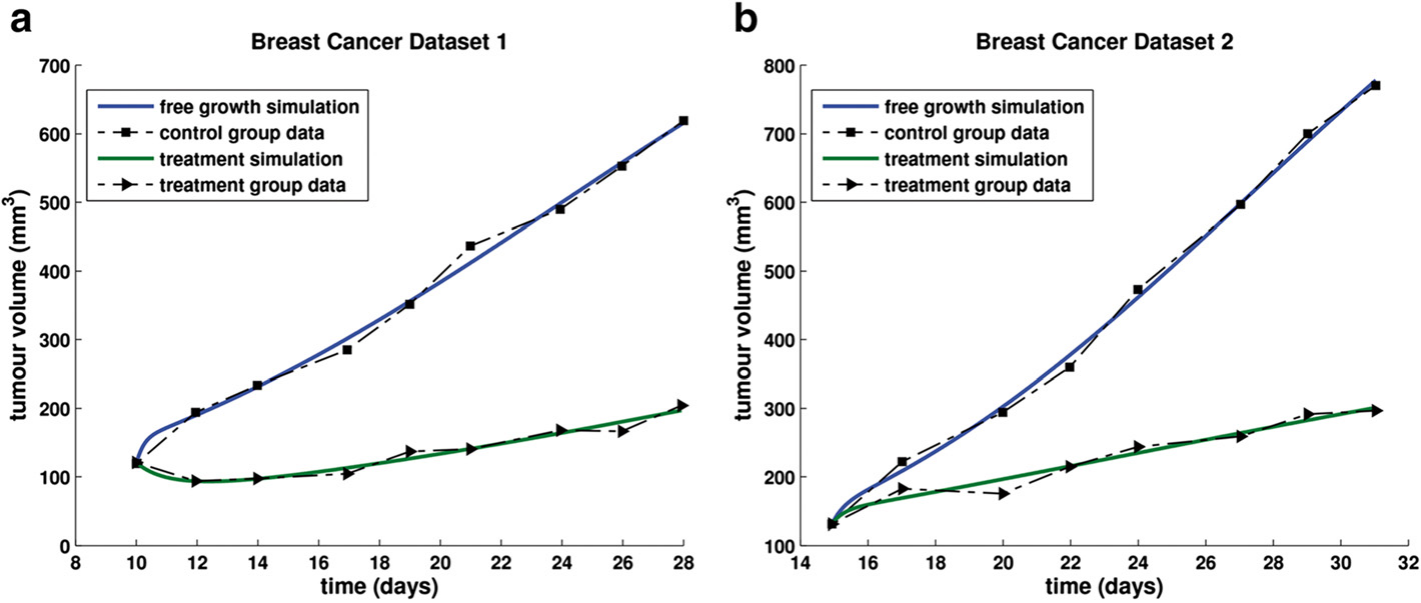
Fitting the model to breast cancer data. Graphical representation of the fitting results of the vascular tumour growth and response to bevacizumab treatment model to experimental data concerning KPL-4 human estrogen receptor-negative breast adenocarcinoma xenografts that were extracted from [60]: **a** In the context of a bevacizumab monotherapy study, **b** In the context of a bevacizumab combination treatment study

*Experiment 1*: In the context of a monotherapy antitumour efficacy study, twenty mice bearing KPL-4 human estrogen receptor-negative breast adenocarcinoma xenografts were divided into a vehicle and a treatment group (10 animals per group) dosed with bevacizumab at 5 mg/kg twice weekly intraperitoneally [60]. The results of the local fitting are presented in Table 4 and Fig. 11.

**Table 3:** Free growth-related parameters involved in the calculation of tumour volume plateau and range of values that yield biologically acceptable tumour behaviour

Parameters	Range of values	Units
*c*	[0.933 – 87.3]	day^−1^
*d*	[0.000585 - 0.054]	day^−1^ · mm^−2^

*Experiment 2*: A second dataset of twenty mice bearing KPL-4 human estrogen receptor-negative breast adenocarcinoma xenografts has been divided into a vehicle and a treatment group (10 animals per group) dosed with bevacizumab at 5 mg/kg twice weekly intraperitoneally [60]. The specific experiment has been conducted in the context of a combination treatment antitumour efficacy study but only the free growth – related and bevacizumab monotherapy-related data were exploitable by the model. The results of the local fitting are presented in Table 4 and Fig. 11.

**Fig. 12:**
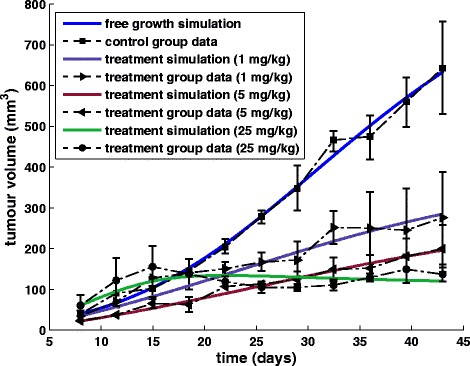
Fitting the model to lung cancer data. Graphical representation of the fitting results of the vascular tumour growth and response to bevacizumab treatment model to experimental data concerning lung tumour xenografts [61]

*Experiment 3*: Four groups of athymic mice bearing H226 xenografts (lung cancer cells) were treated with IgG (control) or 3 dose levels of bevacizumab (1, 5 and 25 mg/kg intraperitoneally) twice weekly for a total of 9 doses (3 mice per group) [61]. The results of the local fitting are presented in Table 5 and Fig. 12.

**Table 4:** Parameter values that have been specified by locally fitting the vascular tumour growth model to experimental data extracted from [60]

Study	Experimental group	*λ*_1_	*c*	*d*	*β*	*p*	*K*_*0*_	RMSE	FOO
		day^−1^	mg/day · mm^3p^ · kg	day^−1^ · mm^−2^	mm^3p^	-	mm^3^	mm^3^	
Dataset 1	Control	4.7129	0.1519	0.0015	-	-	159.4476	10.7552	3.31 · 10^−2^
	Treatment	0.6701	0.0991	0.0015	1	4.0606	76.0920	7.0026	1.5 · 10^−3^
	(5 mg/kg)								
Dataset 2	Control	0.1340	1.1503	0.0088	-	-	650	13.3087	1.12 · 10^−2^
	Treatment	0.1049	0.5236	0.0088	1.4456	3.8916	358.2054	11.7711	6.72 · 10^−5^
	(5 mg/kg)								

First-order optimality (FOO) is a measure of the closeness of the solution to the optimum

**Fig. 13:**
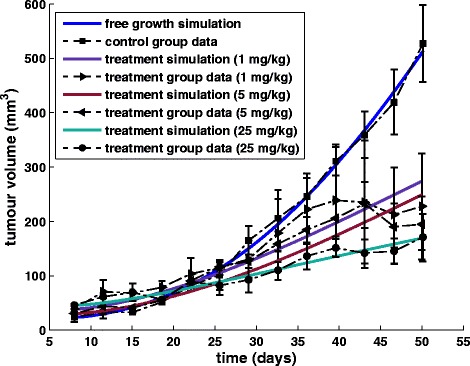
Fitting the model to head and neck cancer data. Graphical representation of the fitting results of the vascular tumour growth model to experimental data concerning head and neck tumour xenografts [61]

*Experiment 4*: Four groups of athymic mice bearing SCC1 xenografts (head and neck cancer cells) were treated with IgG (control) or 3 dose levels of bevacizumab (1, 5 and 25 mg/kg intraperitoneally) twice weekly for a total of 9 doses (3 mice per group) [61]. The results of the local fitting are presented in Table 5 and Fig. 13.

**Fig. 14:**
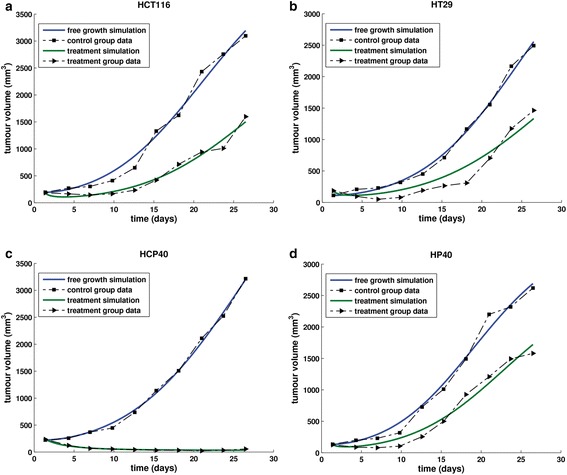
Fitting the model to colon cancer data. Graphical representation of the fitting results of the vascular tumour growth model to the experimental data that were extracted from [62]. Local fitting using xenograft mouse tumour models of colon cancer cell lines **a**. HCT116 **b**. HT29 **c**. HCP40 **d**. HP40

*Experiment 5*: Xenograft mouse tumour models of colon cancer cell line HCT116 were divided in a control and a treatment group. The treatment group received 15 mg/kg of bevacizumab intraperitoneally twice weekly for four weeks, while the control group received the same amount of nonspecific murine IgG antibody [62]. The results of the local fitting are presented in Table 6 and Fig. 14a.

**Table 5:** Parameter values that have been specified by locally fitting the vascular tumour growth model to experimental data extracted from [61]

Cell line	Experimental group	*λ*_1_	*c*	*d*	*β*	*p*	*K*_*0*_	RMSE	FOO
		day^−1^	mg/day · mm^3p^ · kg	day^−1^ · mm^−2^	mm^3p^	-	mm^3^	mm^3^	
H226 (lung cancer cells)	Control	0.1028	0.7264	0.0075	-	-	208.6162	16.7273	2.57 · 10^−1^
	Treatment 1	0.1028	0.3982	0.0075	1	4.9963	183.4523	22.2226	10^−3^
	(1 mg/kg)								
	Treatment 2	0.1028	0.3214	0.0075	1	5.6544	118.7044	8.0860	1.12 · 10^−4^
	(5 mg/kg)								
	Treatment 3	0.1028	0.1735	0.0075	1	5.6024	320.1562	21.1671	3.31 · 10^−4^
	(25 mg/kg)								
HSCC1 (head and neck cancer cells)	Control	0.0444	3.3548	0.0198	-	-	23.6087	10.2691	9.5 · 10^−2^
	Treatment 1	0.0444	1.4969	0.0190	1	5.0006	38.9545	27.8009	1.2 · 10^−3^
	(1 mg/kg)								
	Treatment 2	0.0444	1.4895	0.0190	1	6.0014	30.6914	27.8653	6.88 · 10^−4^
	(5 mg/kg)								
	Treatment 3	0.0444	0.8864	0.0190	1	5.5002	46.0371	9.7061	7.65 · 10^−5^
	(25 mg/kg)								

First-order optimality (FOO) is a measure of the closeness of the solution to the optimal

*Experiment 6*: Xenograft mouse tumour models of colon cancer cell line HT29 were divided in a control and a treatment group. The treatment group received 15 mg/kg of bevacizumab intraperitoneally twice weekly, while the control group received the same amount of nonspecific murine IgG antibody [62]. The results of the local fitting are presented in Table 6 and Fig. 14b.

*Experiment 7*: Xenograft mouse tumour models of colon cancer cell line HCP40, a cell line derived by exposing HCT116 cells to sublethal periods of hypoxia three times weekly for 40 exposures, were divided in a control and a treatment group. The treatment group received 15 mg/kg of bevacizumab intraperitoneally twice weekly, while the control group received the same amount of nonspecific murine IgG antibody [62]. The results of the local fitting are presented in Table 6 and Fig. 14c.

*Experiment 8*: Xenograft mouse tumour models of colon cancer cell lines HP40, a cell line derived by exposing HT29 cells to sublethal periods of hypoxia three times weekly for 40 exposures, were divided in a control and a treatment group. The treatment group received 15 mg/kg of bevacizumab intraperitoneally twice weekly, while the control group received the same amount of nonspecific murine IgG antibody [62]. The results of the local fitting are presented in Table 6 and Fig. 14d.

The agreement of numerical simulations with experimental data supports the validity of the underlying model. At this point it should be stressed that since the model presented focuses only on bevacizumab monotherapy, the body of experimental data that could be used in order to test the model was quite limited. Therefore, we had to make use of this limited experimental data body in order to fit the model. Taking this into account, the results and conclusions presented are to be viewed as preliminary signs of evidence rather than definitive validated knowledge. Additionally, it should be borne in mind that due to the complexity of cancer models, the scarcity and the low quality of experimental/clinical data, the potential danger of overfitting leading to over-optimistic results is very common.

All results were obtained with a maximum termination tolerance on the function value equal to 10^−6^. Following the evaluation of normalized root mean square error (see NRMSE column in Table 7) questions have emerged about a few cases where a non-satisfying fit has been obtained (e.g. experiment 3: cell line H226, treatment group, dose level 25 mg/kg and experiment 4: cell line HSSC1, treatment group, dose level 5 mg/kg). Among possible causes could be human error in digitization of the experimental data with the use of the PlotDigitizer software and the locality of the selected fitting method. In particular, local solvers such as the one used in this case provide the user with a local minimum depending on the selected starting point, but not necessarily the best or global minimum. This means that had the solver been initialized at different parameter values than the selected ones [31], better results could have come up. This claim is also supported by the fact that the aforementioned fitting difficulty seems to mostly occur in the therapeutic module of the model and especially in cases of higher dosage administration (5–25 mg/kg) where a greater perturbation of free growth parameter values is expected.

**Table 6:** Parameter values that have been specified via locally fitting the vascular tumour growth model to experimental data extracted from [62]

Cell line	Experimental group	*λ*_1_	*c*	*d*	*β*	*p*	*K*_*0*_	RMSE	FOO
		day^−1^	mg/[day · mm^3p^ · kg]	day^−1^ · mm^−2^	mm^3p^	-	mm^3^	mm^3^	
HCT116	Control	0.2362	0.4884	0.0017	-	-	194.5946	121.7580	7.35 · 10^−1^
	Treatment	0.2362	0.3881	0.0017	1	5.7777	0.6	74.8503	6.61 · 10^−2^
	(15 mg/kg)								
HCP40	Control	0.1603	0.5036	0.0013	-	-	223.3251	47.8814	1.30 · 10^−1^
	Treatment	0.1233	0.0186	0.0013	0.9062	12.4022	17.2066	9.3102	8.76 · 10^−5^
	(15 mg/kg)								
HT29	Control	0.2138	0.5061	0.0017	-	-	111.5735	58.1716	2.17 · 10^−1^
	Treatment	0.2138	0.3783	0.0017	1	6.2774	0.6	129.8810	1.5 · 10^−3^
	(15 mg/kg)								
HP40	Control	0.2966	0.5056	0.0022	-	-	134.0895	100.5007	3.77 · 10^−1^
	Treatment	0.2966	0.4356	0.0022	1	4.5576	23.0272	93.2413	1.33 · 10^−1^
	(15 mg/kg)								

First-order optimality (FOO) is a measure of the closeness of the solution to the optimum

It should be noted however, that potential non-uniqueness of a solution could suggest over-parameterization of a model. In fact, due to the incomplete quantitative understanding of the behaviour of neoangiogenesis, pure tumour growth and their interaction and at the same time to the need for a more detailed description of certain fundamental qualitative mechanisms which collectively constitute the nature of phenomenon of cancer, some degree of over-parameterization seems to be currently unavoidable. In this case, identifiability analysis could determine whether the parameters involved in the system are uniquely estimable from the experimental data and if not, whether the nonidentifiability is structural or practical, i.e. it can be overcome with the use of a different experimental dataset. A discussion on identifiability analysis can be found in [67–71]. However, it should be noted that as at this point in time the exact boundaries of the region of multidimensional parameter space yielding biologically acceptable tumour volume behaviour have not yet been determined, this kind of analysis is rather premature. In particular, even if the system was proved nonidentifiable it could still be valid as in real-life applications, such as the one studied in this paper, what we seek for is not a solution unique for the parameter space in its entirety, but a solution unique in a specific parameter subspace that includes valid parameter values for e.g. a human cancer patient. Even if more than one equally good solutions come up for a specific dataset, the availability of further clinical data which would allow us to impose constraints on the optimal solution narrowing down the potential solutions could be of vital importance. Eventually, functional data such as positron emission tomography (PET) or functional magnetic resonance imaging (fMRI) and histopathological data with the help of which the modeller can gain insight into the case-specific appropriate parameter space will be able to assist the determination of a personalized and as such unique solution.

In order to address a part of the aforementioned limited discrepancies, multiple actions need to be taken. First of all, extra experimental datasets should be extracted from relevant literature, ideally concerning actual clinical patients and directly available so as not to risk human error due to the digitization of the experimental data with the use of PlotDigitizer software, in order to elucidate the exact reasons of the occurrence of these incidents and act accordingly. Another obviously significant task would be to conduct global fitting of the vascular tumour growth model so as to attempt to determine solutions for the problematic cases of fitting encountered so far. Currently, the Multistart solver in Global Optimization Toolbox is used in conjunction with Matlab functions lsqnonlin, fmincon and fminsearch.

## Conclusions

The main focus of this work is to study and extend a continuous approach describing vascular tumour growth under angiogenic signalling presented in [43]. To this end, the pharmacokinetic properties of bevacizumab have been incorporated into the model and the monotherapy effect has been simulated for the specific angiogenic inhibitor, which probably is the most popular representative of the wider family of antiangiogenic agents in clinical practice. Additionally, the model has been modified in order to reproduce the asymptotic behaviour of tumour volume in the theoretical case of a total destruction of the tumour neovasculature. The results produced by the model have been thoroughly studied and reveal qualitative similarities with experimental observations. An initial exploratory parametric analysis has been conducted, in order to gain insight into the free growth behaviour of a vascularized tumour. In this context, the role of endogenous proangiogenic and antiangiogenic factors in tumour evolution has been examined as well as their combined effect on the eventually attained tumour volume plateau. Insight into the significance of a tumour size - specific treatment planning has been revealed. The conducted parameter analysis suggests that an initial chemotherapeutic intervention when the tumour is sufficiently small, followed by antiangiogenic treatment when the tumour reaches a critical size would be the most efficient treatment strategy. This does not contradict the current clinical practice suggesting treatment combining antiangiogenic treatment with chemotherapeutic agents but highlights the potential significance of a prior chemotherapeutic intervention. Further investigation is necessary to confirm this claim. Furthermore, local fitting of the vascular tumour growth model to actual experimental data concerning the *in vivo* study of solid tumours has been performed so as to acquire an initial confirmation that the underlying mathematical model [31, 43] is actually tailored to the nature of the problem under study. The global fitting of the model is currently underway. The successful reproduction of the results of a series of *in vivo* experiments in mice treated with bevacizumab lends support to the underlying model.

A meaningful extension of this work would be to simulate the effect of combination treatment consisting of both antiangiogenic treatment and cytotoxic agents. In this case, the model should be modified so as to account for the synergistic effect of the concomitant administration of antiangiogenic and chemotherapeutic treatment. Another interesting modification, would be to consider a saturation effect based on the existence of the concept of optimal biological dose reported in relevant literature. Finally, the consideration of diverse cell populations would allow the modeller to exploit potentially available histological data concerning cell composition of the tumour and link it with the response to a specific treatment scheme.

At this point it should be noted that stochasticity appears to be an important characteristic of malignant tumour growth and response to treatment. Despite the fact that the model presented is a deterministic one, it could also be used in a stochastic context as follows: in order to compare candidate treatment schemes and/or schedules *in silico*, several possible combinations of parameter values lying around their apparently most probable estimates have to be constructed so as to cover the abstract parameter space as best as possible. Code executions have to be performed for all these selected parameter combinations. If, for example, the clinical question addressed is “Which one of the two candidate treatment schedules denoted by I and II is the most promising for a given patient?” simulations have to be run for both schedules I and II and for all parameter value combinations selected. If based on the simulation predictions schedule I outperforms schedule II for a sufficiently large percentage of the total parameter combinations considered, say 90 %, then there is ground to suggest adoption of schedule I [72]. It should also be mentioned that a number of predominantly stochastic tumour growth models [73], vascular tumour growth models [74] and angiogenesis models [75] have also appeared in literature. However, the majority of such models has not been developed bearing targeted antiangiogenic treatment in mind.

Our ultimate goal is to simulate the spatiotemporal response of clinical tumours to various treatment schemes and schedules in the patient individualized context. This extremely challenging task entails the exploitation of multiscale clinical data, for the purpose of conducting thorough fitting and validation studies of the model and obtaining parameter estimates representative of the human patient. An invaluable help toward obtaining patient-specific predictions is expected to be provided by the identification of potential biomarkers of bevacizumab response, emerging from numerous clinical trials. It should be noted that the work presented is also positioned within the large scale integrated project CHIC [Computational Horizons In Cancer (CHIC): Developing Meta- and Hyper-Multiscale Models and Repositories for In Silico Oncology (FP7-ICT-2011-600841)]. The latter aims at developing cutting edge ICT tools, services and secure infrastructure to foster the development of elaborate and reusable integrative models (hypermodels) and larger repositories so as to demonstrate benefits of having both the multiscale data and the corresponding models readily available. In this context, the presented model constitutes one of the component models of the CHIC model repository, with the ultimate goal of being combined with other CHIC models to form clinically-relevant hypermodels.

## Reviewers’ comments

### Reviewer's report 1*:* Dr Leonid G Hanin, Department of Mathematics, Idaho State University

**General Comments**

1. The results of the paper seem to suggest that under antiangiogenic treatment the volume of a tumor will always eventually increase and reach a high-level plateau. Then why does one need such treatment? Isn’t the goal of antiangiogenic therapy to shrink the tumor to the avascular level and induce dormancy?

Authors’ response*: Based on the originally expected effect of antiangiogenic treatment, the rather obvious goal would be to render a tumour avascular and induce quiescence. However, it should be noted that following years of antiangiogenic agents’ use in the clinical setting, several possible explanations of the actual effect of this kind of treatment have been proposed. Apart from the partial regression of tumour vasculature (von Baumgarten et al. 2011), one of the most widely accepted theoretical explanations is based on the effect of vascular normalization (Jain, 2003). The latter may lead to a more efficient access of the* chemotherapeutic drug *to tumour cells which is administered in conjunction with antiangiogenic treatment. It is also pointed out that the antiangiogenic agent is in fact cytotoxic per se for certain tumour types (Drevs, 2008). Therefore, the final treatment outcome may prove to be a quite complex one.*

*Additionally, it should be stressed that certain theoretical explanations proposed up to now appear to be at least partly mutually conflicting. Therefore, further experimental, clinical and in silico investigation is needed in order to clarify the open issues.*

*Regarding the eventual tumour repopulation, this appears to be inevitable in cases where even a single cell has survived the treatment. However, even a transient tumour volume decrease could render an inoperable tumour to an operable one, offering the patient a chance to eventually cure the disease. Finally, even though we agree with Dr Hanin in that ideally, the goal of antiangiogenic treatment would be to render the tumour avascular, it should be taken into account that even if in several cases this kind of therapy does not induce tumour volume decrease at all, due to intrinsic or evasive tumour resistance, the valuable effect of tumour growth delay is still obtained. From the clinician’s viewpoint, this effect is considered valuable because not only it extends the patient’s lifespan but it also enhances the patient’s quality of life.*

Drevs J: VEGF and angiogenesis: implications for breast cancer therapy. *European Journal of Cancer Supplements* 2008, 6:7–13.

Jain RK: Molecular regulation of vessel maturation. *Nature Medicine* 2003, 9:685–693.

von Baumgarten L, Brucker D, Tirniceru A, Kienast Y, Grau S, Burgold S, Herms J, Winkler F: Bevacizumab Has Differential and Dose-Dependent Effects on Glioma Blood Vessels and Tumor Cells. *Clinical Cancer Research* 2011, 17:6192–6205.

2. P. 22. The fact that in the absence of treatment tumor volume V may decrease (that is, V may become larger than the carrying capacity K) is somewhat troubling. This probably means that model (1)-(2) is inadequate and should be changed through a more careful study of pro- and anti-angiogenesis forces. Overcoming this problem by imposing a restriction on parameter values seems artificial.

Authors’ response*: This kind of behaviour occurs in two cases. First, if the user selects biologically invalid initial values (V*_*0*_*, K*_*0*_*) i.e. K*_*0*_ *< V*_*0*_*. Second, if the user selects a biologically irrelevant combination of parameter values involved in the model. For example, by setting parameter c as equal to zero, one creates a tumour with no endogenous angiogenic stimulation and thus a self-regressing tumour. However, in both cases in which the model yields a behaviour that is obviously incompatible with clinical reality, the input would be just nonsensical since it would not correspond to a biologically valid behaviour. These are restrictions imposed by the biological reality and not artificial constraints. The model is only a tool and the quality of input always determines the quality of output. In an eventual future integration of the model into a clinical decision support system appropriate input checkers will be included in order to minimize the chances for nonsensical input.*

3. I do not understand the motivation behind local parameter optimization. When parameters are fit to the observed time course of tumor volume they ideally should be optimized globally (under natural constraints like positivity) and the initial values of model variables should also be subjected to global optimization. What prevents the authors from doing this?

Authors’ response*: We completely agree with Dr Hanin in that global fitting of the model is clearly superior to local fitting, especially if one takes into account the frequent presence of multiple local minima rendering the outcome of a local investigation uncertain. However global fitting requires the investigation throughout a finite parameter space. In our case, the physical constraint of non-negativity alone could not sufficiently narrow the range of parameter values. This difficulty still leaves the search of parameter space in its entirety an extremely computationally challenging task. Indeed, although the relevant code had already been developed by us, we did not proceed with its use as the initial results were discouraging in terms of computational demands. Hence, instead of constraining the problem in a random and arbitrary way, as a first step we performed the investigation of the local behaviour of the model around the available reference values. Of course, as we also stress in the manuscript, all results and conclusions drawn in the context of the local fitting of the model are to be viewed as preliminary signs of evidence rather than definitive validated knowledge.*

4. Some parameters of models (1)-(2) and (5)-(6) are non-identifiable. For example, in the absence of treatment (I = 0) the models depend on the ratio c/*α*. Also, in the case *p* = 0 considered in the paper the treatment model depends in reality not on three parameters c, *α*, *β* but rather on two combinations, c(β + 1) and α(β + 1), of these parameters. Therefore, model parameters cannot be fixed arbitrarily and varied or optimized independently. In particular, the choice α = β = 1 made in the paper forces the authors to deal from the outset with a sub-optimal model. Model non-identifiability and the resulting overparameterization may explain various troubles that the authors had with parameter estimation. To be useful, the models should be formulated in terms of identifiable combinations of the original parameters. For a general discussion of structural identifiability for both deterministic and stochastic models, the authors are referred to the following paper:

L.G. Hanin (2002), Identification problem for stochastic models with application to carcinogenesis, cancer detection and radiation biology, Discrete Dynamics in Nature and Society 7(3):177–189.

Authors’ response*: Regarding Dr Hanin’s point about model sub-optimality, it should be noted that both α and β were set equal to 1 as in (Poleszczuk et.al* [43]*), only for demonstration purposes in the context of the Results and Discussion subsection entitled “**Indicative tumour growth patterns**”. For the needs of the automated fitting process, parameter β has been allowed to fluctuate around the initial value β = 1. Thus, all β values appearing in Tables*3*,*4*, and*5*have been estimated via the least squares solver. The reason why many of those values remain equal to 1 is that high values of parameter p have rendered parameter β insensitive to perturbations of its value. Thus, the least squares solver minimized the cost function using the rest of the parameters that were more sensitive comparing to β.*

*Regarding the identifiable parameter combinations, we agree with Dr Hanin that this kind of analysis should be performed and we plan to conduct it in our future work.It is noted that the work presented is only an initial approach to the highly complex and demanding problem of simulating vascular tumour growth under bevacizumab treatment and adapting the simulation model to real animal and/or clinical data. The available data is very limited and mostly incomplete. Therefore, the manuscript aims at contributing to paving the way for the development of a clinically useful model and its in vivo fitting rather than providing an extensively validated and ready to use model. In this context, the effect of each individual parameter corresponding to a given bioprocess has been studied in order to gain some initial insight into the physical behaviour and the physical understanding of the model. As we also mention above, we plan to continue the exploration of the parameters’ effects and eventual interdependences in future work. The same applies to the extension of our initial fitting methodology from estimating individual parameter values to estimating values of identifiable parameter combinations.*

*Regarding identifiablility analysis, as we also mention in our response to Prof Radivoyevitch, it is a valuable tool to evaluate the predictive power of the model since the outcome of the model prediction is dependent on the identifiability of the parameters involved. However, in our case, identifiability analysis was considered premature, in the sense that the exact boundaries of the multidimensional parameter subspace yielding biologically acceptable tumour volume behaviour have not been yet determined. Hence, even if the system proved to be nonidentifiable we think it could still be valid for the following reason. In real-life applications such as the one studied in this manuscript, what we actually seek for is not a unique solution for the parameter space in its entirety, but a unique solution in a specific parameter subspace that includes valid parameter values for e.g. a human cancer patient. Finally, we have included the source suggested by Dr Hanin in the relevant discussion in p.34 of the subsection entitled “**Local fitting results**”.*

Poleszczuk J, Bodnar M, Foryś U: New approach to modeling of antiangiogenic treatment on the basis of Hahnfeldt et al. model. *Math Biosci Eng* 2011, 8:591–603.

5. The transition from model (3)-(4) in the original paper to model (7)-(8) in its revision was triggered by my comment about inconsistency of model (3)-(4). Because Biology Direct is an open review journal, this should probably be acknowledged.

Authors’ response*: We thank Dr Hanin for his important remark. We completely agree. Our updated approach along with an explicit acknowledgement to Dr Hanin is included in the subsection entitled “**A continuous approach to modelling the mechanism of action of antiangiogenic treatment applied on a vascularized tumour**” which is part of the section “**Methods**.” More precisely, the following text has been inserted “Prompted by a remark made by Dr. Leonid Hanin in his capacity as a reviewer of the present manuscript, we have proceeded to a correction of the asymptotic behaviour of the system as the drug concentration tends to infinity* (*I*(*t*) → ∞)*. According to our initial gross assumption and in agreement with the asymptotic behaviour of the ODE model developed by Hahnfeldt et al. (1999) and Poleszczuk et. al* [43]*, the tumour volume tends to zero as I*(*t*) → ∞*. This appears to be acceptable as a first approximation. However, we have adopted Hanin’s suggestion to take into account the refined mathematical observation that as I*(*t*) → ∞ the tumour volume should tend to V*. V* corresponds to the critical avascular tumour volume.” In addition, Dr Hanin is specially acknowledged for the same remark in the Acknowledgements section.

Hahnfeldt P, Panigrahy D, Folkman J, Hlatky L: Tumor development under angiogenic signaling: a dynamical theory of tumor growth, treatment response, and postvascular dormancy. *Cancer Res* 1999, 59:4770–4775.

Poleszczuk J, Bodnar M, Foryś U: New approach to modeling of antiangiogenic treatment on the basis of Hahnfeldt et al. model. *Math Biosci Eng* 2011, 8:591–603.

6. Eqs. (3) and (4) were derived in [28] on certain biological grounds. I do not see why the same logic that led to relationships between V and K also holds for the quantities V-V* and K-V*.

Authors’ response: We thank Dr Hanin for his remark. Indeed, the modified equations are not derived on the exact same basis as the initial ones. The slightly modified assumption behind the updated equations suggests that the amount of angiogenic stimulators secreted by the tumour does not actually depend on the entire tumour volume V but, instead, on the part of tumour volume that has been developed following the angiogenic switch i.e. on the quantity V-V*. This is biologically plausible, if we take into account that the angiogenic switch, when the tumour has already reached the critical size V*, is actually the moment at which the tumour begins to overexpress angiogenic stimulators (Hanahan et al. [49]). In other words, once in the vascular phase during which the model is applicable, the part of the tumour volume developed prior to the triggering of the angiogenic switch does not contribute to the secretion of proangiogenic factors and subsequently to the growth of carrying capacity. Similarly, angiogenic inhibitors are assumed to depend on the quantities V-V* and K-V*.

*We have added a discussion regarding the impact that our modification has on the initial assumptions in p.14 of the Methods subsection entitled “**A continuous approach to modelling the mechanism of action of antiangiogenic treatment applied on a vascularized tumour**.”*

Hanahan D, Weinberg RA: Hallmarks of Cancer: The Next Generation. *Cell* 2011, 144:646–674.

7. Is it true that every solution of system (3)-(4) converges to the steady-state solution? Could there be oscillatory solutions? These are important questions that should be addressed from the very beginning.

Authors’ response*: Poleszczuk and his colleagues (2011) have analysed the dynamics of the core of the model for the case of constant treatment. According to this analysis, there is a sufficient condition for the existence of a unique steady state in ℝ*_+_^2^*for the ODE system. Clearly, there may also be a unique steady state solution in ℝ*_+_^2^*, with no fulfilment of the specific condition. Poleszczuk et al. have proved that if the positive steady state for the system is unique in ℝ*_+_^2^*then it is globally stable in ℝ*_+_^2^*. In particular, according to Poincare- Bendixson theorem any solution tends to either a steady state or to a closed orbit. However, with the use of Dulac-Bendixson criterion they conclude to the non-existence of a closed orbit.*

*Although we agree with Dr Hanin in the significance of the qualitative study of the dynamical system, for the initial local exploration of the physical behaviour of the model (via parameter analysis and local fitting) which was the aim of this work, we felt that a bifurcation analysis was out of scope for the time being. However, we intend to conduct this kind of analysis in the near future.*

Poleszczuk J, Bodnar M, Foryś U: New approach to modeling of antiangiogenic treatment on the basis of Hahnfeldt et al. model. *Math Biosci Eng* 2011, 8:591–603.

8. The structure of the treatment term in Eq. (2) should be explained. In particular, what is the role of parameter p and why is it set equal to 0?

Authors’ response*: We thank Dr Hanin for his suggestion. Regarding the structure of the treatment term, it is noted that it came up in (Poleszczuk et al.* [43]*) based on the solution of a diffusion equation for the concentration of angiogenic stimulators. This in turn was based on specific approximations and underlying assumptions that are listed in the manuscript. There is a qualitative description of all significant steps of the modification performed by Poleszczuk et al. in the Methods subsection entitled “**A continuous approach to modelling the mechanism of action of antiangiogenic treatment applied on a vascularized tumour**” and specifically in p.12. Regarding the mathematical details, the reader is referred to (Poleszczuk et al.* [43]*).We chose not to repeat the presentation of all calculations to avoid overburdening the reader with information that is thoroughly explained in already published work.*

*The p parameter was introduced in the model through the fifth assumption in p. 10: “The change of drug concentration inside the tumour caused by the dysfunctional vasculature is governed by the proportionality to a bounded and decreasing function of tumour volume.” In particular, the function that was used by Poleszczuk et al. was of the form*$\frac{1}{\beta +{V}^p},p\ge 0$*. Hence, parameter p reflects the extent of the abnormal phenotype of tumour vasculature.*

*Parameter p was set equal to 0 only for the presentation needs of indicative results for the following two reasons:****a.****0 was one of the values used in (Poleszczuk et al.* [43]*)****b.****0 was the most suitable value for demonstration purposes in the sense that it created the more pronounced therapeutic effect for the specific tumour (characterized by the λ*_*1*_*, c and d values in (Hahnfeldt et al. 1999) and the specific treatment scheme which is widely applied in the context of in vivo experiments. This fact allowed a straightforward observation of all significant qualitative features of the simulation.*

*Finally, an updated, more specific description of the parameters that were introduced in the system by Poleszczuk et al. i.e. α, β and p, can be found in Table*1*.*

Hahnfeldt P, Panigrahy D, Folkman J, Hlatky L: Tumor development under angiogenic signaling: a dynamical theory of tumor growth, treatment response, and postvascular dormancy. *Cancer Res* 1999, 59:4770–4775.

Poleszczuk J, Bodnar M, Foryś U: New approach to modeling of antiangiogenic treatment on the basis of Hahnfeldt et al. model. *Math Biosci Eng* 2011, 8:591–603.

9. P. 36. The authors concluded that when the tumor is small it should be first treated with cytotoxic chemotherapy and when it reaches some critical size an antiangiogenic treatment should be given. This recommendation seems to be self-defeating, for chemotherapy will shrink the tumor even further and so the conditions for antiangiogenic treatment will not be met.

Authors’ response*: What was meant by this statement is that at the timepoint of diagnosis, when the clinician needs to decide on the therapeutic strategy, the optimal treatment modality should be selected in a size-specific way. For example, in the case where a tumour is in its initial stage of vascular development the clinician should bear in mind that cytotoxic treatment would be more efficient comparing to antiangiogenic treatment. However, it also holds that even though in the short term chemotherapy will indeed shrink the tumour, eventually, due to acquired resistance mechanisms, the tumour will probably start growing again. Hence, the conditions for antiangiogenic treatment will eventually be met unlessof course the tumour is surgically excised.*

10. P. 37, 2 paragraph. What is described as stochastic approach is not stochastic at all!

Authors’ response*: The sense in which the term “stochasticity” is used in our extended approach outlined in the Conclusions section is the following: The new “multi-thread” model for a given patient is made up of a number of “one-thread” models along with their input parameter combinations. These parameter combinations are created by randomly (stochastically) selecting values from the corresponding value distributions. Distribution moments such as the mean value and the standard deviation in the case of a normal distribution are provided as input to the “multi-thread”. The use of random (or more precisely pseudorandom generators) renders this multi-thread model stochastic.*

**Technical Comment**s

1. What is *λ*1? Is it the intrinsic rate of cancer cell division or the net proliferation rate?

Authors’ response*: Parameter λ*_*1*_*is the net proliferation rate reflecting both the intrinsic rate of cancer cell division and cancer cell death induced by non-metabolic causes such as the directly cytotoxic effect of bevacizumab. Cell death related to the metabolic conditions in tumour microenvironment is accounted for through the vascular compartment i.e. Eq.*6*.*

2. What is tumor vasculature normalization?

Authors’ response*: Vasculature normalization is a hypothesis made by Jain in 2001, stating that antiangiogenic treatment improves both functionally and morphologically the abnormal structure of tumour vessels. This transient effect, known as normalization window, results in a more normal and organized vasculature network and thus allows chemotherapy to access a larger part of the tumour and function more effectively. This hypothesis is consistent with the bulk of clinical data suggesting a synergistic effect between antiangiogenic treatment and chemotherapy. We have added a few lines elaborating on tumour vasculature normalization in p.6 of the**Background**section.*

*“In particular, the antiangiogenic treatment effect is believed … sites due to the high abnormality of tumour vessels.”*

3. P. 6, end of paragraph 1. The meaning of “tumor immunity” is unclear. Do you mean tumor immunogenicity?

Authors’ response*: Tumour immunity is the precise term utilized in (Sato* [25]*). What the specific source considers as enhancement of tumour immunity is a better and easier access of leukocytes into the tumour parenchyma due to antiangiogenic treatment - induced vascular normalization. Hence, antiangiogenic treatment also functions as an amplifier of the reaction of the immune system to the tumour. However, we agree with Dr Hanin that using the widely accepted terminology the term immunogenicity would better express the phenomenon. Therefore, the latter has been used in the revised manuscript.*

Sato Y: Persistent vascular normalization as an alternative goal of anti-angiogenic cancer therapy. *Cancer Sci* 2011, 102:1253–1256.

4. Exponent 2/3 is missing in Eqs. (6) and (8).

Authors’ response*: We have inserted the missing exponents*.

5. Why do Eqs. (10)-(11) not account for the initial dose accumulation?

Authors’ response*: Eqs. (*10*)-(*11*) constitute part of the solution of the mass balance equations corresponding to the two-compartmental model for a single intravenous infusion. As we explain in p. 17 of the Methods subsection entitled “Inclusion of bevacizumab pharmacokinetic properties” the pharmacokinetic effect of multiple drug doses is computed internally in the code, by superpositioning all drug concentration curves at each simulation timepoint so as to sum the contribution of current drug dose with the one of previous administrations. Hence, the initial dose accumulation which can be observed in Fig.*4*is accounted for in the code of the pharmacokinetic model.*

6. P. 25. “A closer inspection of Fig. 4a reveals that each time the curve K(t) intersects curve V(t), the monotonicity of the function V(t) alters.” This is obvious from Eq. 3.

Authors’ response*: This is an observation enabling the reader to grasp the theoretical concept of carrying capacity which is known only to an audience familiar with population dynamics modelling. For further clarification, we have added the sentence “This behaviour that can be easily noticed from Eq.*3*reflects the fact that carrying capacity is actually defined as the maximal tumour volume that the current vascular system can support.” following the above statement.*

7. P. 26, Parameter analysis results. The concept of doubling time pertains to exponential growth. For non-exponential growth, it is not a well-defined quantity.

Authors’ response*: We agree with Dr Hanin. However, the term “doubling time” was used in the manuscript in a pointwise sense, aiming at communicating to the reader a physical interpretation of the role of λ*_*1*_*parameter. In particular, taking into account that a Gompertzian curve can be considered as a piecewise exponential curve, the doubling time can be determined in a pointwise mode i.e. at each timepoint. To improve this sentence, we have rephrased it as follows.*

*“Equation (*7*) shows that the Gompertzian growth constant λ*_*1*_*is the only parameter that affects tumour growth rate independently of the angiogenic compartment, making it thus a direct modulator of the doubling time characterizing the simulated tumour at each timepoint.”*

**Comments on Style**

1. The paper should be translated from “international sciencespeak” into normative English. Reading some parts of the paper was quite painful. Many words are misused, and many expressions and phrases are awkward or grammatically incorrect. They are far too numerous to list. Monstrous sentences like “To this end, a mechanistic model monitoring both the tumour and vascular compartment while addressing the targeted nature of the specific kind of treatment will serve as a vehicle to valuable insight into anti-angiogenic treatment mode of action” on p. 9 or “The subsequent step following the development of the continuous approach describing vascular tumour growth under angiogenic signalling and its extension via the inclusion of bevacizumab pharmacokinetic properties is corroborating that the mathematical structure of the model reflects the nature of the problem under investigation” on p. 20 should be rephrased and broken down into simpler sentences or dropped altogether. The authors are advised to consult a native English speaker.

Authors’ response*: We have rephrased and shortened many sentences and we have improved the language of the manuscript.*

2. To prop up their statements about cancer biology, the authors should refer the reader to biological rather than mathematical papers (see ref. [3, 4, 7, 8, 46]). Similarly, reference to a paper in nursing (ref. [15]) is probably not the best choice.

Authors’ response*: We have added more suitable sources*.

3. Some references are not quoted sequentially. For example, ref. [41] on p. 7 is missed as are refs [59] and [60] on p. 23. Also, ref. [71] does not seem to appear anywhere in the text.

Authors’ response*: We have corrected the references.*

4. The paper contains several repetitions as well as lengthy and unnecessary discussions. The results section can be compressed. Also, the number of tables and figures is too large. Some tables can be combined and some omitted because they duplicate results presented graphically.

Authors’ response*: Repetitions have been removed and multiple discussions have been shortened. We have merged Tables*1*,*2*and*7*into one table. However, we fear that by trying to further condense these items and the Results section important information might be rendered relatively unclear.*

**Table 7:** Normalized root-mean-square error calculated for each experimental dataset utilized in the context of model fitting

Cell line	Reference	Experimental group	Range of experimental values	RMSE	NRMSE
KPL-4 (Bevacizumab monotherapy study)	[60]	Control	500.25	10.76	2.15 %
		Treatment	83.23	7.00	8.41 %
		(5 mg/kg)			
KPL-4 (Bevacizumab combination treatment study)	[60]	Control	639.84	13.31	2.08 %
		Treatment	165.70	11.77	7.10 %
		(5 mg/kg)			
H226	[61]	Control	603.07	16.73	2.77 %
		Treatment 1	241.23	22.22	9.21 %
		(1 mg/kg)			
		Treatment 2	177.17	8.09	4.57 %
		(5 mg/kg)			
		Treatment 3	76.32	21.17	27.74 %
		(25 mg/kg)			
HSCC1	[61]	Control	504.05	10.27	2.04 %
		Treatment 1	188.87	27.80	14.72 %
		(1 mg/kg)			
		Treatment 2	164.08	27.87	16.99 %
		(5 mg/kg)			
		Treatment 3	125.12	9.71	7.76 %
		(25 mg/kg)			
HCT116	[62]	Control	2902.71	121.76	4.19 %
		Treatment	1411.98	74.85	5.30 %
		(15 mg/kg)			
HCP40	[62]	Control	2992.56	47.88	1.60 %
		Treatment	178.66	9.31	5.21 %
		(15 mg/kg)			
HT29	[62]	Control	2381.34	58.17	2.44 %
		Treatment	1276.84	129.88	10.17 %
		(15 mg/kg)			
HP40	[62]	Control	2484.60	100.50	4.04 %
		Treatment	1454.25	93.24	6.41 %
		(15 mg/kg)			

5. P. 38. An advertisement of the activities of the authors’ workplace should not be a part of scientific paper.

Authors’ response: *Having taken into account this comment we have rephrased the paragraph under consideration as follows: “Our ultimate goal is to simulate the spatiotemporal response of clinical tumours to various treatment schemes and schedules in the patient individualized context. This extremely challenging task entails the exploitation of multiscale clinical data, for the purpose of conducting thorough fitting and validation studies of the model and obtaining parameter estimates representative of the human patient. An invaluable help toward obtaining patient-specific predictions is expected to be provided by the identification of potential biomarkers of bevacizumab response, emerging from numerous clinical trials. It should be noted that the work presented is also positioned within the large scale integrated project CHIC [Computational Horizons In Cancer (CHIC): Developing Meta- and Hyper-Multiscale Models and Repositories for In Silico Oncology (FP7-ICT-2011-600841). The latter aims at developing cutting edge ICT tools, services and secure infrastructure to foster the development of elaborate and reusable integrative models (hypermodels) and larger repositories so as to demonstrate benefits of having both the multiscale data and the corresponding models readily available. In this context, the presented model constitutes one of the component models of the CHIC model repository, with the ultimate goal of being combined with other CHIC models to form clinically-relevant hypermodels.” Please note that no working affiliations appear in this updated version of the paragraph.*

6. Ref. [4] does not include page numbers.

Authors’ response: *This has been addressed.*

7. Check the title of ref. [6].

Authors’ response: *This was corrected.*

Quality of written English: Not suitable for publication unless extensively edited.

## Final comments

**Reviewer Recommendation Term:** Endorse publication

**Quality of written English:** Acceptable

**Reviewer summary:** In response to my comments the authors included additional explanations and caveats into the paper making it clear that several aspects of their work are preliminary. The style of the paper has improved. There are still a few minor stylistic imperfections present; hope they can be addressed by the journal’s technical editor. My final minor technical comments are given in the Comments to Authors.

**Reviewer recommendations to authors:** The new revision represents an improvement both in terms of substance and style. I think that another round of revision is unnecessary.

### Reviewer's report 2*:* Prof Tomas Radivoyevitch, Case Western Reserve University, USA*.*

Relative to the landmark work of Hahnfeldt et al. [28] on which this work is based, moving toward least squares estimates of parameters is a step in the right direction as it yields 95 % confidence intervals (CI) of the parameter estimates.

Least squares estimation is done here for 4 parameters, but CI were not provided. Presumably CI were not shown because some had upper limits of infinity, but knowing which parameters could not be identified, and for those that could, the extent to which they could (based on how large the upper limits are), would have been informative.

In the third paragraph prior to Conclusions it is mentioned that different initial conditions for the optimization lead to different answers. I see this as indicating over-parameterization that cannot be overcome by starting in different negative log-likelihood valleys. The model should be parsimonious enough for fitting to be fairly robust to fairly reasonable initial parameter values. If this were achieved, initial values would not be an explanation of poor fit. I also would not blame PlotDigitizer. Sloppy experiments and the model missing a concept/other parameters are two other possibilities, but the model is already over-parameterized, so perhaps living with some correlated residuals is OK for now. Recent papers on identifiability of biomathematical ODEs include:

1. Raue, A., Kreutz, C., Theis, F.J. & Timmer, J. Joining forces of Bayesian and frequentist methodology: a study for inference in the presence of non-identifiability. Philosophical transactions. Series A, Mathematical, physical, and engineering sciences 371, 20110544 (2013).

2. Kreutz, C., Raue, A. & Timmer, J. Likelihood based observability analysis and confidence intervals for predictions of dynamic models. BMC Systems Biology 6, 120 (2012).

3. Raue, A., Becker, V., Klingmuller, U. & Timmer, J. Identifiability and observability analysis for experimental design in nonlinear dynamical models. Chaos 20, 045105 (2010).

4. Raue, A. et al. Structural and practical identifiability analysis of partially observed dynamical models by exploiting the profile likelihood. Bioinformatics 25, 1923–9 (2009). Implementation. Simulations in R with ODEs in C are fast enough to be nested in likelihood calculations. The model of Hahnfeldt et al. is available in such C code in the files philMod.c and philModAE.c in http://epbi-radivot.cwru.edu/EPBI473/files/wk13tumorTherapy. Also available in this folder are scripts that use this code to produce maximum likelihood estimates (including 95 % CI from Hessians); these scripts were written in 2005 (for the R package odesolve) and could be streamlined today using the R packages bbmle (for CI and profiles) and deSolve (which adds conveniences that extend odesolve).

Authors’ response*: We thank Prof Radivoyevitch for his remarks and suggestions on our work.*

*The sentence “In particular, local solvers such as lsqnonlin provide the user with a local minimum depending on the selected starting point, but not necessarily the best or global minimum.” was meant to explain the way that a local solver works. What we simply intended to state was that having in most cases searched through a very specific “neighbourhood” of the parameter space (we perturbed the parameters to be estimated around reference values) and given the locality of the fitting method used, we cannot be sure that there is not a better solution somewhere else in the parameter space.*

*Of course, we agree with Prof Radivoyevitch that identifiability analysis is a valuable tool to evaluating the predictive power of the model since the outcome of the model prediction is dependent on the identifiability of the parameters involved. However, in our case, identifiability analysis was considered premature, in the sense that the exact boundaries of the multidimensional parameter subspace yielding biologically acceptable tumour volume behaviour have not been yet determined. Hence, even if the system proved to be nonidentifiable it could still be valid, as in real-life applications, such as the one studied in this paper, what we actually seek for is not a solution unique for the parameter space in its entirety, but a solution unique in a specific parameter subspace that includes valid parameter values for e.g. a human cancer patient.*

*A paragraph has been added, stating the possibility of over-parameterization of the model, explaining the significance of identifiability analysis and also our rationale. See page 34 “However, potential non-uniqueness of a solution… human cancer patient.”*

### Reviewer's report 3: Dr Lutz Edler, German Cancer Research Center, Germany

The authors have addressed all my comments in the reply letter and implemented changes in response to a larger part of the many comments, accounting also the limitations set by the journal (e.g. not having figures and tables near the text they belong to), This correspondence together with the substantially revised text should suffice the paper now for the benefit of the engaged reader enthusiastic for modeling carcinogenesis, even if I liked to see methods and results better seperated.

In conclusion the paper is ready for publication from this perspective. If the authors have the option to polish English language and to some extent alos grammar, they should do so. However, I also see the trade off between such editing and the delay of publication. Therefore "acceptable" is indicated.

Quality of written English: Acceptable

Authors’ response*: We thank Dr Edler for his remarks. We have further improved the language of the manuscript*.

## Abbreviations

ODE: ordinary differential equations
PDE: partial differential equations
VEGF: vascular endothelial growth factor
